# Physicochemical characterization and genotoxicity of the broad class of carbon nanotubes and nanofibers used or produced in U.S. facilities

**DOI:** 10.1186/s12989-020-00392-w

**Published:** 2020-12-07

**Authors:** Kelly Fraser, Vamsi Kodali, Naveena Yanamala, M. Eileen Birch, Lorenzo Cena, Gary Casuccio, Kristin Bunker, Traci L. Lersch, Douglas E. Evans, Aleksandr Stefaniak, Mary Ann Hammer, Michael L. Kashon, Theresa Boots, Tracy Eye, John Hubczak, Sherri A. Friend, Matthew Dahm, Mary K. Schubauer-Berigan, Katelyn Siegrist, David Lowry, Alison K. Bauer, Linda M. Sargent, Aaron Erdely

**Affiliations:** 1 Health Effect Laboratory Division, National Institute for Occupational Safety and Health, 1095 Willowdale Rd, MS-2015, Morgantown, WV 26505-2888 USA; 2 West Virginia University, Morgantown, WV USA; 3 Health Effects Laboratory Division, National Institute for Occupational Safety and Health, Cincinnati, OH USA; 4 West Chester University, West Chester, PA USA; 5 RJ Lee Group, Monroeville, PA USA; 6 Repiratory Health Division, National Institute for Occupational Safety and Health, Morgantown, WV USA; 7 Division of Field Studies Evaluation, National Institute for Occupational Safety and Health, Cincinnati, OH USA; 8 International Agency for Research on Cancer, Lyon, France; 9 Department of Environmental and Occupational Health, University of Colorado Anschutz Medical Campus, Aurora, CO USA

## Abstract

**Background:**

Carbon nanotubes and nanofibers (CNT/F) have known toxicity but simultaneous comparative studies of the broad material class, especially those with a larger diameter, with computational analyses linking toxicity to their fundamental material characteristics was lacking. It was unclear if all CNT/F confer similar toxicity, in particular, genotoxicity. Nine CNT/F (MW #1–7 and CNF #1–2), commonly found in exposure assessment studies of U.S. facilities, were evaluated with reported diameters ranging from 6 to 150 nm. All materials were extensively characterized to include distributions of physical dimensions and prevalence of bundled agglomerates. Human bronchial epithelial cells were exposed to the nine CNT/F (0–24 μg/ml) to determine cell viability, inflammation, cellular oxidative stress, micronuclei formation, and DNA double-strand breakage. Computational modeling was used to understand various permutations of physicochemical characteristics and toxicity outcomes.

**Results:**

Analyses of the CNT/F physicochemical characteristics illustrate that using detailed distributions of physical dimensions provided a more consistent grouping of CNT/F compared to using particle dimension means alone. In fact, analysis of binning of nominal tube physical dimensions alone produced a similar grouping as all characterization parameters together. All materials induced epithelial cell toxicity and micronuclei formation within the dose range tested. Cellular oxidative stress, DNA double strand breaks, and micronuclei formation consistently clustered together and with larger physical CNT/F dimensions and agglomerate characteristics but were distinct from inflammatory protein changes. Larger nominal tube diameters, greater lengths, and bundled agglomerate characteristics were associated with greater severity of effect. The portion of tubes with greater nominal length and larger diameters within a sample was not the majority in number, meaning a smaller percentage of tubes with these characteristics was sufficient to increase toxicity. Many of the traditional physicochemical characteristics including surface area, density, impurities, and dustiness did not cluster with the toxicity outcomes.

**Conclusion:**

Distributions of physical dimensions provided more consistent grouping of CNT/F with respect to toxicity outcomes compared to means only. All CNT/F induced some level of genotoxicity in human epithelial cells. The severity of toxicity was dependent on the sample containing a proportion of tubes with greater nominal lengths and diameters.

## Introduction

The evaluation of the potential toxicity of carbon nanotubes and nanofibers (CNT/F) began in the early 2000’s [1–4]. The general outcomes of toxicity studies to date indicated that pulmonary exposure to CNT/F was capable of inducing inflammation, fibrosis, cancer, immunosuppression, and adverse cardiovascular and neurological outcomes *in vivo* [5–15]. Studies of key importance also confirmed that certain CNT/F were able to translocate from the lung to lung-associated lymph nodes as well as systemic tissues [5, 16–19]. These results raised justifiable concerns regarding potential human health effects, especially in the occupational workforce, and prompted the need to design and conduct epidemiological studies. While the latency needed for clinical symptoms has not ended based upon other fiber toxicity models, as the average worker handling CNT/F has had just short of a decade of cumulative exposure, evidence suggests exposure-related effects primarily consisting of measures of inflammation, oxidative stress, and immunosuppression [20–28]. The outcomes were generally mild with no consistent pattern of effect among studies. Evidence of CNT/F in the sputum was observed and a considerable number of workers, approximately 70%, were subjected to dermal exposure [21, 29]. The National Institute for Occupational Safety and Health (NIOSH) established a recommended exposure limit (REL) of 1 μg/m^3^ as an 8-h time-weighted average of respirable elemental carbon, a surrogate for CNT/F, following background correction for ambient elemental carbon [30]. Dahm *et al*. (2018) found that U.S. companies can, in fact, maintain the 1 μg/m^3^ REL, as 93% of respirable measures were below the REL from 214 collected samples at 12 different facilities [29], although historically, and globally, this has not always been the case [31]. More recently, potential adverse effects of the inhalable fraction, including airway fibrosis and bronchiolitis obliterans [32, 33], have been recognized. The inhalable fraction was often significantly greater than the respirable fraction by 4 times and 29% of the inhalable samples in U.S. facilities were greater than 1 μg/m^3^ [29]. Recently, the International Agency for Research on Cancer (IARC) classified one multi-walled carbon nanotube (MWCNT), the Mitsui-7 or MWCNT-7, as possibly carcinogenic to humans (Group 2B) [34]. There was insufficient evidence to classify all other CNT/F. The 2020–2024 Report of the Advisory Group to Recommended Priorities for the IARC Monographs indicates MWCNT as a high priority to be ready for evaluation within five years [35]. In summary, 1) *in vivo* studies indicated a significant hazard potential of CNT/F, 2) evidence exists of human exposure and health effect, 3) exposure can be controlled at recommended levels, 4) reevaluation for carcinogenicity is imminent, and 5) recommendations to fill toxicity knowledge gaps by examination of a broader class of CNT/F was warranted.

Our group recently conducted a cross-sectional study to evaluate exposure and potential associated health effects in workers handling CNT/F [20–22, 29]. From these studies, which evaluated 12 different facilities, and the years of ongoing exposure assessment of more than 20 facilities [36, 37], it was clear that a wide variety of CNT/F were being produced or utilized by primary and secondary manufacturers. The production of CNT/F continues to increase, and new high-volume applications are being evaluated, especially in the construction sector. The global CNT market is expected to grow from approximately USD 4.5 billion to USD 10 billion by 2023 and USD 15 billion by 2026 with a compound annual growth rate of 16%. The primary question arising from a commercialization, industrial hygiene, and research perspective was whether all as-produced CNT/F materials confer similar toxicity. In controlled studies, differing physicochemical characteristics of CNT, such as length, diameter, functionalization, or surface coating in turn altered the *in vivo* pulmonary toxicity profile [32, 38–49]. To date, very few studies simultaneously compared a broad class of as-manufactured CNT and linked the relationship between physicochemical characteristics and toxicity endpoints.

In this current series of studies, with guidance from extensive facility exposure assessment [29, 36, 37], we selected six MWCNT and two carbon nanofibers (CNF), collectively termed CNT/F, either manufactured or handled by U.S. companies, to evaluate four primary parameters of toxicity using *in vitro* and *in vivo* studies. Specific CNT/F types were selected to be broadly representative of those to which U.S. workers may be commonly exposed. The parameters included genotoxicity, inflammation, pathology, and extrapulmonary translocation. CNT/F selection was initially based on provided company diameter. Nominal tube diameter was the simplest way to delineate samples for testing and previous studies indicate a changing toxicity profile with increasing diameter (or rigidity) [16, 39, 45, 50]. The selected materials ranged from 6 to 150 nm in diameter according to company specifications. Determining the materials to test according to diameter, other key physicochemical characteristics also were expected to vary, such as length (5–200 μm), thus providing a proper representation of the CNT/F material class. A seventh MWCNT, Mitsui-7/MWCNT-7, was added as a benchmark material given the IARC carcinogenicity classification and the large amount of historical toxicity data available for the four parameters of interest. Of the materials selected, four MWCNT had reported company diameters smaller than the benchmark material, and two MWCNT and two CNF had diameters larger than the benchmark material. In the few comparative studies that examined multiple different materials, the larger diameter materials were not evaluated [39, 41, 45].

For this section of the evaluation of CNT/F toxicities, all materials were extensively characterized, and genotoxicity, one of the four primary parameters of toxicity, was evaluated *in vitro*. Analyses included physical dimension, residual metal catalysts, dustiness, density, charge, acellular reactivity, surface area, endotoxin and PAH impurities, thermogravimetric analysis, and hydrodynamic diameter in suspension. Prevalence and forms of bundled agglomerates were also characterized as exposure assessment indicated that agglomerates, not singlets or individual fibers, represent the majority of particles in personal breathing zone samples in workplaces [36]. Human bronchial epithelial cells were treated with CNT/F to determine cell viability, inflammation, oxidative stress, micronuclei formation, and DNA double-strand breakage. Computational modeling was applied to physicochemical characteristics alone, and in conjunction with toxicity outcomes. The modeling created clustering by material, as well as response, to evaluate the relationship between physicochemical characteristic(s) and various toxicity endpoints.

## Results/discussion

**Fig. 1:**
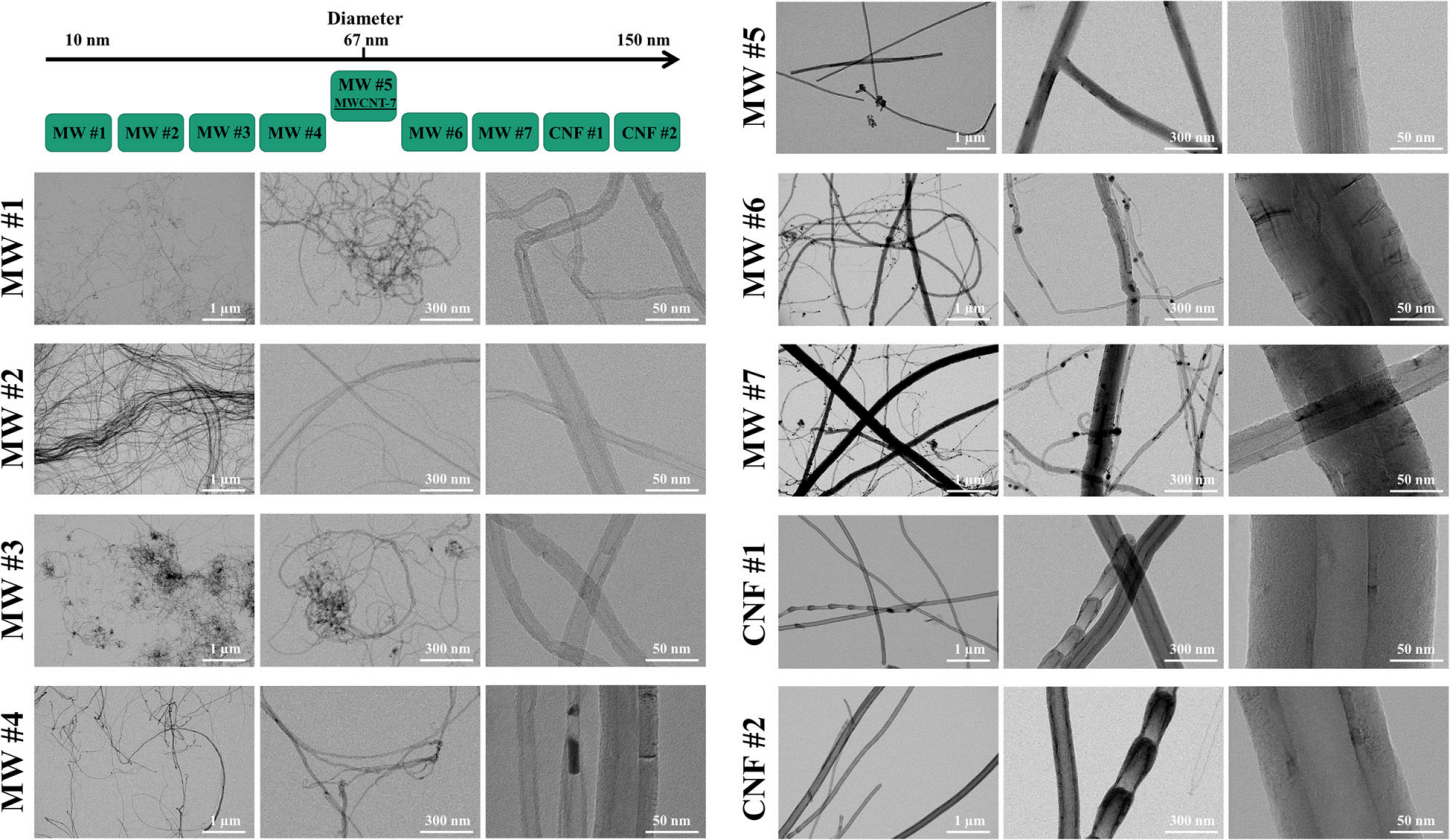
Schematic of material diameter and TEM images of CNT/F. Materials selection was based upon company reported diameter ranging from 6 to 150 nm in diameter to ensure a full range of materials were included in this study and the material arrangement is depicted in the upper left corner. These materials were identified as MW #1–7 and CNF #1–2. A well-studied benchmark material, MWCNT-7/Mitsui-7, was included in this study as MW #5. Materials were dispersed in isopropanol and placed onto a TEM grid to measure physical dimensions. Representative images of each material were selected with scale bars representing 1 μm, 300 nm, and 50 nm from left to right

**Fig. 2:**
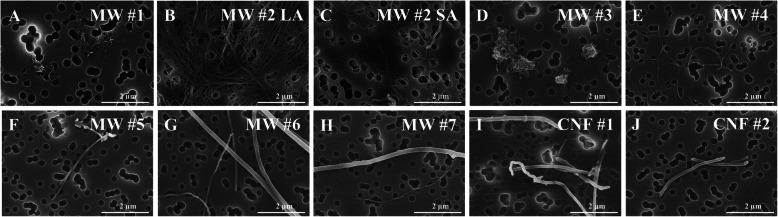
Representative scanning electron microscopy images of CNT/F in DM to measure two-dimensional agglomerate sizes. LA = large agglomerates and SA = small agglomerates

**Fig. 3:**
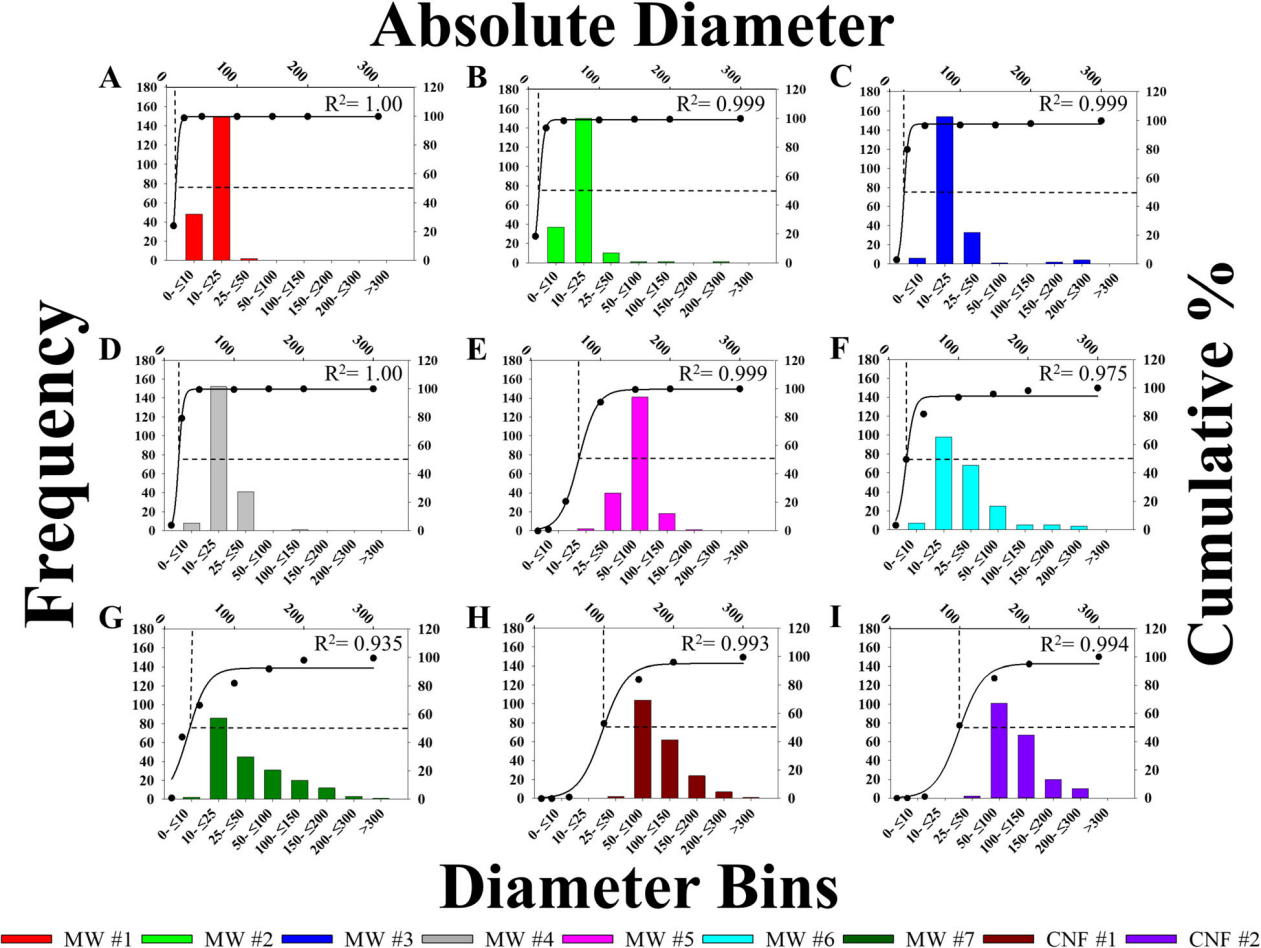
Distributions of CNT/F diameter. Particles were binned according to size along the lower x-axis with frequency on the left y-axis. Additionally, percentage of accumulation is graphed on the right y-axis with the absolute diameter along the upper x-axis. The overlay line was 3 parameter sigmoidal curve of best fit with the point of 50% accumulation indicated with dotted lines. Sizes are for particles in isopropanol suspension

**Fig. 4:**
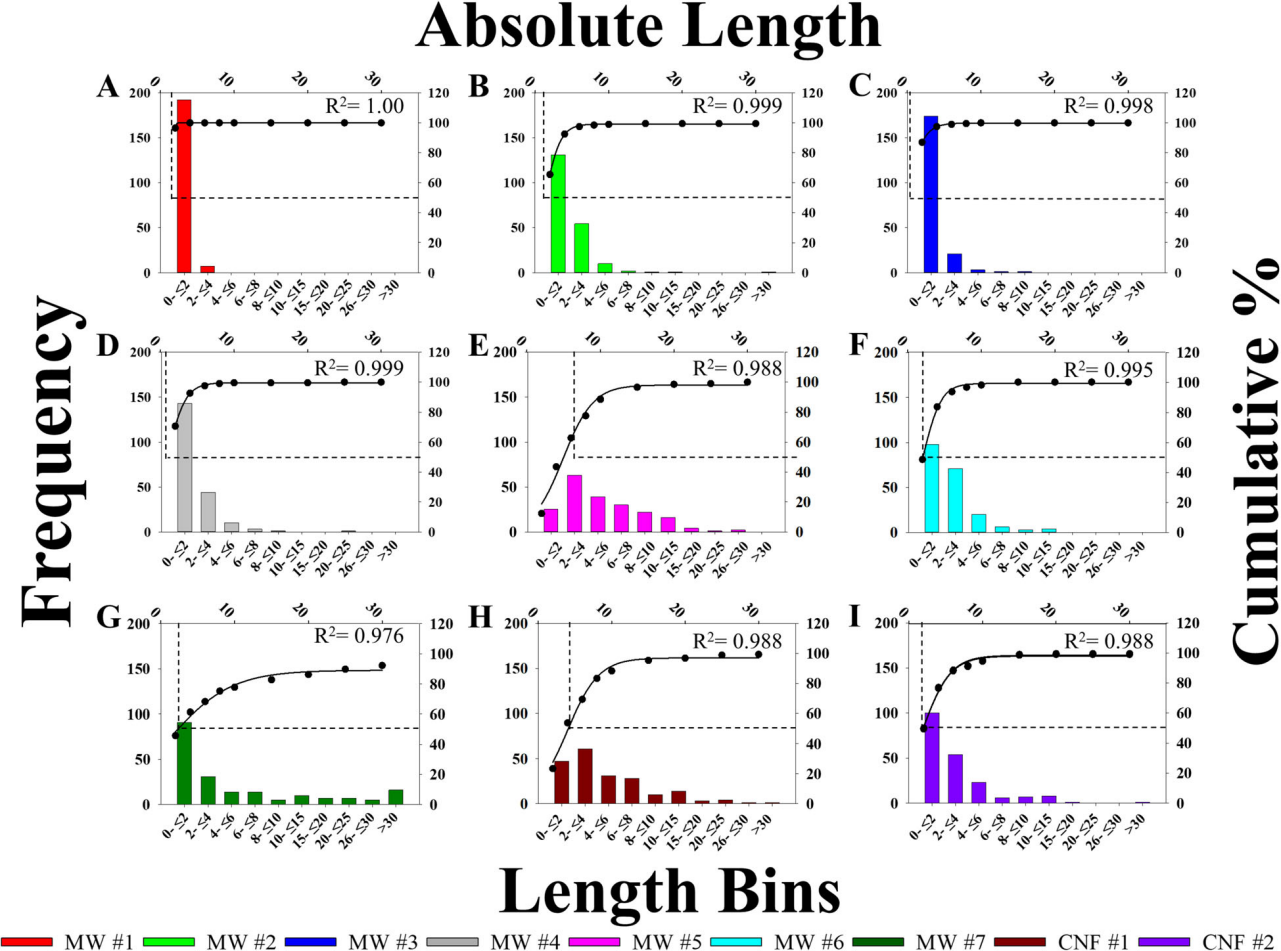
Distributions of CNT/F length. Particles were binned according to size along the lower x-axis with frequency on the left y-axis. Additionally, percentage of accumulation is graphed on the right y-axis with absolute length along the upper x-axis. The overlay line is 3 parameter sigmoidal curve of best fit with the point of 50% accumulation indicated with dotted lines. Sizing was for particles in isopropanol suspension

**Fig. 5:**
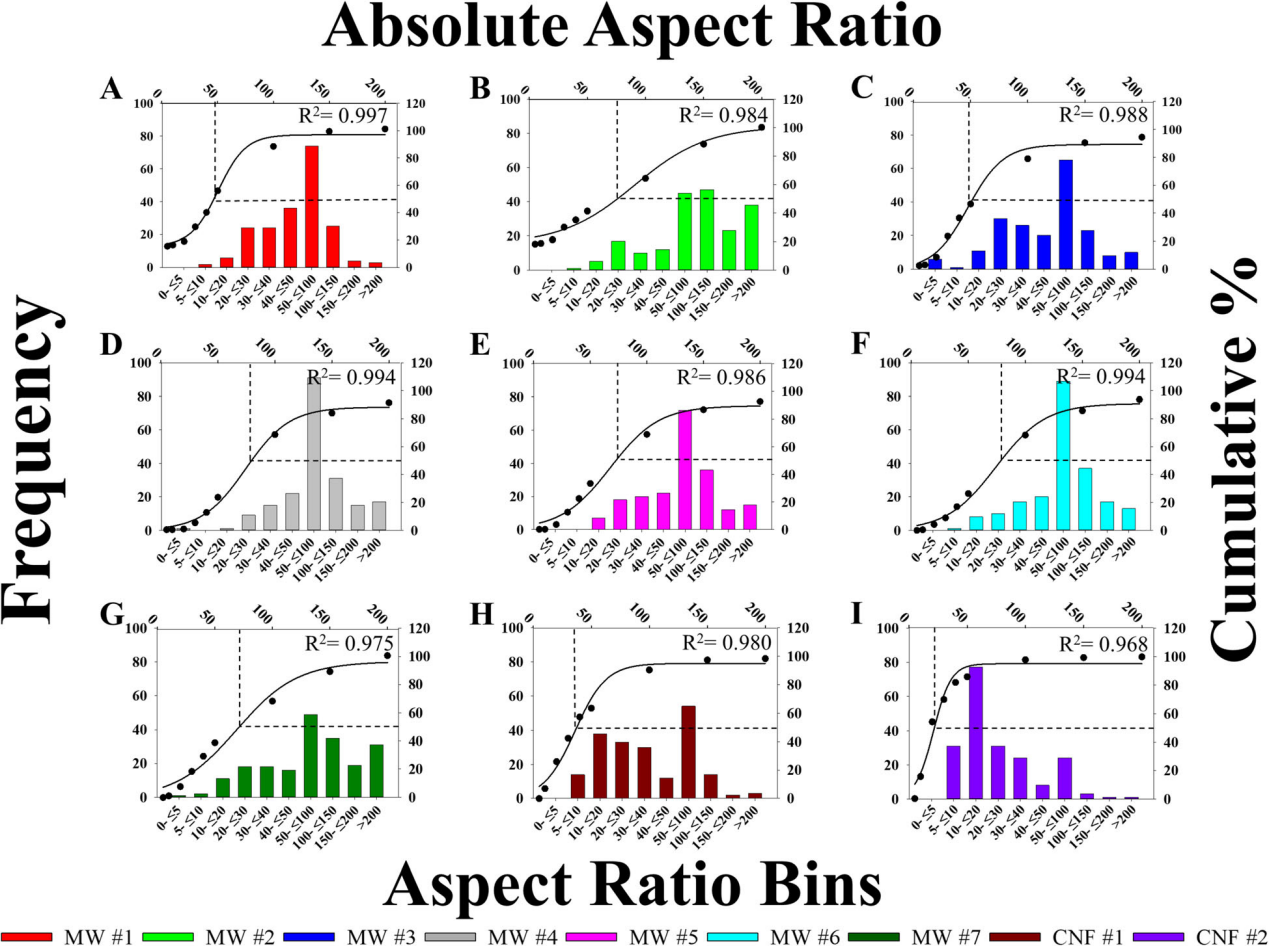
Distributions of CNT/F aspect ratio. Particles were binned according to size along the lower x-axis with frequency on the left y-axis. Additionally, percentage of accumulation is graphed on the right y-axis with absolute aspect ratio along the upper x-axis. The overlay line is 3 parameter sigmoidal curve of best fit with the point of 50% accumulation indicated with dotted lines. Sizing was for particles in isopropanol suspension

Seven MWCNT and two CNF (CNT/F), were arranged according to their diameter as reported by the production facility and are referred to as MW #1–7 and CNF #1–2 (Figs. 1 and 2). The arrangement was designed as the information was readily available from the company and selecting a wide diameter range was necessary to ensure representation of this large class of materials. Furthermore, one material, MW #5, also known as Mitsui-7/MWCNT-7, has been commonly studied and was used as a benchmark material for comparison. All CNT/F were extensively characterized as detailed in Tables 1-3 and Figs. 1-5.

**Table 1:** Physical dimensions of CNT/F dispersed in isopropanol

	MW #1	MW #2	MW #3	MW #4	MW #5	MW #6	MW #7	CNF #1	CNF #2
**Diameter (nm)**									
Company Reported Diameter (nm)	6–9	10	10–15	5–30	N/A	70–80	150	100	150
Geometric Mean (nm ± GSD)	13 ± 1	14 ± 2	20 ± 2	19 ± 1	63 ± 1	28 ± 2	37 ± 2	102 ± 1	103 ± 1
Arithmetic Mean (nm ± SE)	13 ± 0	16 ± 1	26 ± 2	20 ± 1	67 ± 2	38 ± 3	54 ± 4	110 ± 3	110 ± 3
Median	12	14	19	18	63	25	28	98	100
Diameter Range	6–29	6–216	8–275	8–133	21–168	8–218	9–425	40–397	46–263
Normal Distribution	Lognormal								Lognormal
**Length (μm)**									
Company Reported Length (μm)	5	N/A	0.1–10	100	N/A	N/A	N/A	50–200	50–200
Geometric Mean (μm ± GSD)	0.67 ± 1.81	1.34 ± 2.21	1.10 ± 2.00	1.41 ± 1.97	4.39 ± 2.07	2.05 ± 2.53	2.88 ± 4.26	3.64 ± 2.36	2.16 ± 2.31
Arithmetic Mean (μm ± SE)	0.80 ± 0.03	1.79 ± 0.10	1.28 ± 0.07	1.84 ± 0.13	5.62 ± 0.29	3.42 ± 0.37	7.64 ± 0.78	5.23 ± 0.36	3.20 ± 0.28
Median	0.6607	1.5437	1.0148	1.2896	4.547	2.1503	2.3781	3.7273	2.0003
Length Range	0.1–3.6	0.2–50.9	0.1–8.5	0.3–20.6	1.2–25.8	0.3–37.3	0.1–49.1	0.3–37.6	0.4–42.7
Normal Distribution	Lognormal	Lognormal	Lognormal			Lognormal		Lognormal	Lognormal
**Aspect Ratio**									
Aspect Ratio (GeoMean ± GSD)	53 ± 2	96 ± 2	50 ± 2	76 ± 2	69 ± 2	73 ± 2	78 ± 3	36 ± 2	21 ± 2

**Table 2:** Hydrodynamic diameter, zeta potential, and two-dimensional sizing of CNT/F agglomerates dispersed in physiologic dosing media

	MW #1	MW #2 Small Agglomerates	MW #2 Large Agglomerates	MW #3	MW #4	MW #5	MW #6	MW #7	CNF #1	CNF #2
**Spherical Agglomerates**										
% Spherical Agglomerated	87	0	0	83	1	4	0	0	0	0
Spherical Agglomerate Mean Diameter μm ± SE	1.28 ± 0.16	N/A	N/A	0.81 ± 0.10	N/A	N/A	N/A	N/A	N/A	N/A
Spherical Agglomerate Diameter Geometric Mean μm (GSD)	0.96 (2.01)	N/A	N/A	0.66 (1.84)	N/A	N/A	N/A	N/A	N/A	N/A
**Bundled Agglomerates**										
% Bundle Agglomerates/Singlets	13	N/A	N/A	17	99	96	100	100	100	100
Bundle Agglomerate Mean Length μm ± SE	1.90 ± 0.37	3.80 ± 0.38	49.55 ± 3.58	1.11 ± 0.25	3.77 ± 0.35	6.27 ± 0.44	9.47 ± 1.26	11.32 ± 1.08	9.30 ± 1.07	2.96 ± 0.36
Bundle Agglomerate Geometric Mean Length μm (GSD)	1.66 (1.71)	3.09 (1.82)	47.94 (1.31)	0.72 (2.98)	2.92 (2.05)	5.17 (1.94)	5.90 (2.57)	7.91 (2.49)	6.18 (2.49)	2.11 (2.25)
Bundle Agglomerate Mean Diameter μm ± SE	0.38 ± 0.12	0.03 ± 0.00	9.50 ± 2.24	0.03 ± 0.00	0.10 ± 0.01	0.13 ± 0.01	0.08 ± 0.01	0.10 ± 0.01	0.21 ± 0.01	0.12 ± 0.01
Bundle Agglomerate Diameter Geometric Mean μm (GSD)	0.18 (4.20)	0.03 (1.60)	6.99 (2.24)	0.03 (1.56)	0.08 (1.89)	0.11 (1.07)	0.07 (2.09)	0.09 (1.64)	0.19 (1.42)	0.11 (1.63)
**Hydrodynamic** **Diameter (nm)**	660 ± 19	771 ± 33		608 ± 30	478 ± 24	504 ± 15	714 ± 24	652 ± 26	615 ± 19	664 ± 18
**Zeta Potential (pH 7.3)**	−10.4 ± 0.4	−12.1 ± 0.6		−11.1 ± 0.06	−12.0 ± 0.4	−13.5 ± 0.8	−11.8 ± 0.6	−13.2 ± 0.5	−11.1 ± 0.8	−11.3 ± 0.5

**Table 3:** Results of additional particle characterization of CNT/F

	MW #1	MW #2	MW #3	MW #4	MW #5	MW #6	MW #7	CNF #1	CNF #2
**Surface Area** **(m**^**2**^**/g ± SD)**	237.7 ± 1.0	211.9 ± 1.8	218.6 ± 1.2	99.4 ± 1.1	25.2 ± 0.4	25.4 ± 0.4	24.7 ± 0.4	29.4 ± 0.2	18.0 ± 0.2
**Dustiness**									
Dustiness Total (%)	3.8	2.9	0.3	0.5	14.0	0.2	0.2	4.9	ND
Dustiness Respirable (%)	0.84	1.10	0.20	0.20	2.40	0.08	0.09	1.40	ND
**Density**									
Bulk Density (g/cm3)	0.087	0.007	0.082	0.169	0.007	0.075	0.061	0.020	0.032
Tapped Density (g/cm3)	0.119	0.008	0.095	0.222	0.010	0.095	0.073	0.028	0.045
**Endotoxin**	BLD	BLD	BLD	BLD	BLD	BLD	BLD	BLD	BLD
**PAH**	BLD	BLD	BLD	BLD	BLD	BLD	BLD	BLD	BLD
**Metal Catalyst**									
% Fe	0.317	1.725	1.603	3.423	0.270	5.006	6.169	1.168	1.142
% Al	0.310	0.028	2.116	0.019	N/A	0.035	N/A	0.006	0.013
**TGA**									
TGA -Avg onset oxidation, °C	550 ± 2	603 ± 2	575 ± 0	560 ± 2	735 ± 2	581 ± 0	592 ± 0	593 ± 0	694 ± 0
TGA - Mean Residual Ash, %	1.74 ± 0.01	3.98 ± 0.26	8.21 ± 0.26	4.75 ± 0.07	1.11 ± 0.28	7.88 ± 0.15	8.95 ± 0.29	1.79 ± 0.12	2.21 ± 0.16
**Anti-oxidative Capacity**									
%	64.53 ± 23.91	75.41 ± 25.66	76.34 ± 27.37	88.17 ± 28.40	91.49 ± 21.37	84.78 ± 25.74	77.27 ± 19.96	100.12 ± 22.69	99.80 ± 24.19

*BLD* below the level of detection

The typical representation of bundled agglomerates containing tubes/fibers with smaller diameters materials and transitioning to more elongated bundles with tubes/fibers of increasing diameter was readily observed (Fig. 1). Also observed was the range in dimensions that could be present in each sample. For example, MW #2 was a unique material containing two main populations, one with singlets or agglomerates of discrete tubes and the other having highly entangled, cross-linked MWCNT with an average diameter of 7 μm and length of 48 μm as measured by electron microscopy. In contrast, MW #7 had a highly mixed population of diameters that ranged from very thin to very thick with diameters ranging from 9 to 425 nm (Table 1). All CNT/F were extensively characterized and detailed in Tables 1-3 and Figs. 1-5. Of the studies that have simultaneously examined a broad class of CNT/F, a greater proportion of those materials were of diameters at or below MWCNT-7 (mean = 67 nm) [39, 41, 45]. We aimed to extend those studies by encompassing MWCNT with larger diameter tubes and, additionally, by including CNF.

### Nominal tube physical dimensions

The classic fiber paradigm links fiber dimensions and biopersistence with toxicity outcomes. Fibers have been defined by an aspect ratio, or the ratio of particle length to diameter (or width), greater than 3:1 with a length greater than 5 μm and a diameter less than 3 μm [5, 51]. Historically, length, more so than diameter, has been the key consideration in understanding the toxicities induced by high aspect ratio materials. In comparative studies, longer fiber lengths were often associated with greater toxicities of naturally occurring or synthetic fibers [52–62]. Often, materials greater than 5 μm in length were associated with the development of mesothelioma, greater pulmonary biopersistence and particle retention, and greater inflammatory and fibrotic responses. While longer fibers generally confer greater toxicity, short fibers, those less than 5 μm in length, are not without toxicity [63].

The comparison of high aspect ratio CNT/F to asbestos was a natural progression [5, 64–66]. Several comparative studies assessing the effects of length and development of mesothelioma indicated that CNT/F may have similar capabilities to induce adverse effects. General consensus among the literature indicates that longer CNT/F particles were more likely to activate downstream inflammatory cascades, induce fibrogenesis, interrupt macrophage clearance, and were generally more bioactive than short or tightly bundled CNT/F [38, 39, 43, 45, 64, 67–74]. Specific studies on CNT/F diameter, with consistent length, have not been as extensively investigated as a determinant from toxicity outcomes of CNT/F exposure. These studies, sometimes as a comparison of MWCNT to SWCNT, found that increasing diameter can be associated with less toxicity than thinner fibers in terms of inflammation, histopathology changes, alveolar fibrosis, disrupting membrane integrity, and genotoxicity, while other studies link greater diameter to enhanced macrophage interactions, as well as greater apoptosis and inflammation [14, 16, 17, 45, 48, 49, 75–77].

### Nominal tube diameter

As previously noted, company-provided diameter was the initial segregator for deciding which CNT/F to evaluate for toxicity to ensure broad representation of particle sizes. Preliminary evaluations by electron microscopy of the samples also suggested that length was likely to vary with diameter, thereby creating a good representation of the CNT/F class of materials produced and used in U.S. facilities.

To confirm the nominal tube diameters (6–150 nm) reported by the company (Table 1), the CNT/F materials were dispersed in isopropanol and analyzed using scanning transmission electron microscopy (STEM). Two hundred individual tubes for each material were measured and the following parameters were determined: geometric mean, arithmetic mean, range, and median of diameters (Table 1). The samples were further characterized by binning into specific diameter ranges (Fig. 3). From STEM, MW #1–4 had geometric means ranging from 12 to 20 nm (arithmetic means of 13–26 nm) (Table 1). These values were similar to the range of company reported diameters of 6–30 nm. There was a range of 6–275 nm in diameters but very few tubes of MW #1–4 had nominal tube diameters above 50 nm. The geometric mean diameter of the benchmark material, MW #5, was found to be 63 ± 1 nm (arithmetic mean of 67 ± 2 nm) with a range of 21–168 nm, slightly larger than previous reports of a mean of 49 nm [6] but in agreement with other studies [41, 78–80]. MW #6–7 were larger in diameter than MW #1–4 but, on average, smaller than MW #5 (Table 1). Interestingly, while the mean suggests materials smaller in diameter than MW #5, the range and distribution of particles was greatest in the larger size bins (> 150 nm) for MW #6–7 compared to all other MWCNT (Fig. 3). CNF #1 and 2 had diameter geometric means that were similar to each other at 102 ± 1 nm (arithmetic mean 110 ± 3 nm) and 103 ± 1 nm (arithmetic mean 110 ± 3 nm), respectively (Table 1).

It was clear that the central tendency to only include the mean of the distribution of particle dimensions, especially when evaluating MW #6 and #7, did not have enough resolution to fully characterize and distinguish one material from another, a critical factor to understand and model toxicity outcomes based on material properties. The heterogeneity in diameter size distributions of the CNT/F was assessed from the histograms in Fig. 3. The 50% accumulation or cut-off point was determined by curve fitting using the sigmoidal function and is represented by the dashed line with the nominal size value represented by the upper x-axis. The point of 50% accumulation was rapidly achieved for MW #1–4 within the first two bins indicating most particles were less than 25 nm in diameter. Beginning with MW #5, a right shift can be seen, reflecting an increase in diameter. While MW #5 had a significantly larger population of tubes around 64 nm in diameter, the distribution had a smaller range of particle widths; virtually all particles were contained in three bins, compared to MW #6 and 7. While not large in absolute number, subpopulations of larger diameter tubes were found in MW #6 and 7 that were not observed for other MWCNT. CNF #1 and 2 had a similar profile and distribution. Compared to the MWCNT, the shift in 50% accumulation towards larger size bins was more distinguished for the CNF and provided a clear distinction from MW #1–4.

### Nominal tube length

Lengths were not reported by all companies, and those reported had a range of 0.1–200 μm (Table 1). As with diameter, the nominal tube length was determined on tubes/fibers in parallel with diameter to create paired STEM measurements. Two hundred individual tubes or fibers for each material were measured. The summary of length measurements was presented as arithmetic mean, geometric mean, range, median, and binning by specific diameter ranges (Table 1, Fig. 4). MW #1–4 had geometric mean lengths ranging from 0.67–1.41 μm (arithmetic means of 0.80–1.84 μm) (Table 1). MW #1 was the shortest by average length followed by MW #3, with virtually all length values being segregated in the initial bin (0–2 μm) (Fig. 4). MW #5 measured much longer than MW #1–4 at 4.39 ± 2.07 μm (arithmetic mean of 5.62 ± 0.29 μm) with a range of 1.2–25.8 μm. The measured length is consistent with previous reports of MW#5/MWCNT-7 [6]. On average, MW #6–7 were shorter than MW #5 but longer than MW #1–4 (Table 1). The distribution of longer nominal tubes for MW #5–7 was greater than MW #1–4. CNF #1 measured 3.64 ± 2.36 μm (arithmetic mean of 5.23 ± 0.36 μm) in length and CNF #2 was 2.16 ± 2.31 μm (arithmetic mean of 3.20 ± 0.28 μm). The length differences between CNF #1 and 2 was notable as CNF #2 was 40% shorter on average with virtually identical diameters. The arithmetic means of MW #5, #7 and CNF #1 crossed the threshold set by Schinwald *et al*. (2012) (5 μm) for causing acute pleural inflammation [60].

Nominal tube lengths were binned and depicted in histograms found in Fig. 4 with the cut-off points at 50% accumulation indicated in each case. For MW #1–4, almost all (96%) nominal tube lengths were concentrated in the first two size bins. The cumulative distribution of particles and the 50% length accumulation cut-off were shifted to the right for MW #5–7 and CNF #1–2. Overall, bulk samples containing tubes of greater nominal length were more common in MW #5–7, and CNF #1–2, with notably greater length particle populations in MW #5, 7, and CNF #1.

### Aspect ratio

Aspect ratio was a critical measurement considered in the original fiber paradigm. In the 1970s and 1980s, Stanton published his early work linking high aspect ratio materials, particularly glass fibers and asbestos with increased toxicities including lung cancer incidences and mortality [61, 62]. A re-analysis of the research completed in 1980 by Bertrand and Pezerat used multiple regression analysis to conclude that the carcinogenicity of fibers was a continuous spectrum that must include both length and diameter, as a greater aspect ratio can be indicative of greater carcinogenicity [52]. While aspect ratio is an inherent description of length to diameter, the values for each material, including the distribution, were considered for toxicity outcomes.

Individual tube aspect ratio was quantified from STEM measurements as the diameter and length measurements were paired. These measurements were as follows (Geometric Mean ± Geometric standard deviation, GM ± GSD): 53 ± 2, 96 ± 2, 50 ± 2, 76 ± 2, 69 ± 2, 73 ± 2, 78 ± 2, 36 ± 2 and 21 ± 2 for MW #1–7 and CNF #1–2, respectively. MW #4–7 had a slightly higher aspect ratio compared to MW #1 and #3. CNF #1 had a lower aspect ratio compared to all MW due to the notably larger diameter, which was even less for CNF #2 given a similar diameter, but shorter length compared to CNF #1. As with length and diameter, aspect ratios were binned and histograms with corresponding accumulation curves were generated and can be found in Fig. 5. All materials had a wide distribution of aspect ratios. There was a trend for the peak aspect ratio to be from 50 to 100 for all materials except CNF #2. The CNF had a greater leftward distribution with CNF #2 having a significant population of fibers with an aspect ratio of approximately 20. Due to the differences in length, CNF #1 and 2 had notably different distributions of aspect ratios.

### Two-dimensional sizing of agglomerates

Previous studies have considered the role of CNT agglomeration as a determinant of toxicity outcome, particularly within the context of genotoxicity, macrophage recognition, the activation of downstream inflammatory cascades, and pulmonary fibrosis [5, 18, 33, 81–83]. Rod-like and less tangled particles, including singlets, were more likely to influence inflammation histopathology outcomes by inducing more pulmonary fibrosis, and impact extrapulmonary translocation [17, 33, 81]. Furthermore, agglomeration patterns and size are relevant factors in human occupational exposures and respirability [29, 37].

Using SEM images, size measurements of particle agglomerates were completed following dispersion in our physiologic dosing medium, commonly referred to as dispersion medium (DM). We have previously described how the sample preparation mimics collected personal breathing zone samples of workers [32]. Representative SEM images can be found in Fig. 2. Based on the dimensions, particles in this study were categorized into two distinct groups, spherical or bundled agglomerates. Spherical agglomerates were defined as tangles of tubes/fibers that had an aspect ratio of less than 3:1; this convention was adapted from earlier workplace exposure assessment studies [29]. The second category of agglomerates was referred to as “bundles.” These bundled agglomerates were tangles of particle with an aspect ratio greater than 3:1. These bundles varied in the number of tubes/fibers, with some having only a couple. In sizing these structures, the major and minor axes of the bundles were reported as length and diameter, respectively.

MW #1 and #3 were similar with spherical agglomerates composing the bulk (87 and 83%, respectively). A second subpopulation of bundled agglomerates was present, but not dominant. Spherical agglomerates of MW #1 averaged 0.96 ± 2.01 μm (arithmetic mean of 1.28 ± 0.16 μm) in diameter and MW #3 has spherical agglomerates averaging 0.66 ± 1.84 μm (arithmetic mean of 0.81 ± 0.10 μm) in diameter. Bundled agglomerates of MW #1 and #3, which represented less than 20% of the total sample exceeding a 3:1 dimension, had lengths under 2 μm and diameters under 0.4 μm on average.

MW #2 was a unique material that formed quite large agglomerates that were not spherical, but rather interconnected ‘rivers’ of highly entangled cross-linked MWCNT (Fig. 2). These agglomerates were “bundled” agglomerates with a high aspect ratio and two subpopulations were distinguished (Table 2). The large bundled agglomerates averaged 49.55 ± 3.58 μm in length and had an average diameter just under 10 μm. The second subpopulation was found as more loosely bundled, smaller structures, likely agglomerates of singlet tubes as compared to the entangled cross-linked MWCNT. This subpopulation had arithmetic averages of 3.80 ± 0.38 μm in length and 30 nm in diameter. The two populations also highlight that the singlet nominal tube diameter and length of MW #2 was not entirely representative of the material as it did not account for the large bundled agglomerates.

Beginning with MW #4, there was a rather dramatic transition from spherical agglomerates to bundled agglomerates (Table 2). In fact, less than 0–4% of any of MW #4–7 or CNF #1–2 were categorized as spherical agglomerates using our criteria. As the materials increase in diameter, the materials become almost exclusively small bundles and singlets that assume a more classic fiber-like appearance (Fig. 2). The bundled agglomerates, more representative of a fiber-like appearance, were representative of the physical dimensions. Specifically, the length of the bundled agglomerates for MW #4 and CNF #2 were on average 50–75% shorter compared to MW # 5–7 and CNF #1.

### Hydrodynamic diameter and zeta potential

The hydrodynamic diameter, which qualitatively reflects the agglomerated state of the CNT in aqueous solution, was evaluated using dynamic light scattering (DLS). The hydrodynamic diameter ranged between 478 and 771 nm (Table 2). MW #4 was found to have the smallest hydrodynamic diameter, followed by MW #5. Similar values were found for MW #1, 3, 7, and CNF #1 and 2, and MW #2 and MW #6 had the largest hydrodynamic diameters.

Zeta potential, the electrokinetic potential at the interface of the particle surface and aqueous solution, was evaluated by measuring the electrophoretic mobility of the particles in solution by phase analysis light scattering. Zeta potential of a nanomaterial is indicative of its stability in a solution. Minimal differences in zeta potential were observed between these materials (Table 2).

### Surface area

Surface area has been a central measurement for ultrafine particle characterization [84]. Studies have focused on how surface area was a primary determinant of toxicity, especially with metal oxides [85–88]. While surface area is inversely related to nominal tube diameter and decreases with agglomeration, the quantification of the surface area of CNT/F can pose some limitations due to their physical structure [89–91]. For example, the interior space of variable concentric layers paired with porosity, grooves, and other surface topography can lead to variation in measurements of surface area. A few studies have linked increased surface area of CNT/F to more pronounced toxicity outcomes, including genotoxicity and inflammation [45, 81, 92]. In this study, all CNT/F were analyzed using the same methodology, allowing for adequate comparisons between materials (Table 3). The surface areas follow the expected relationship that smaller diameter corresponded to greater surface area on a mass-to-mass basis. MW #1–3 had the greatest surface area. MW #4 was intermediate indicating a transition point in physical dimensions. MW #5–7 and CNF #1 and 2 have the smallest surface area, almost an order of magnitude less than MW #1–3.

### Dustiness

Particle dustiness is a quantification of the tendency of a dry powder to aerosolize, an important aspect for understanding the potential for human occupational exposure. Two independent measurements, total and respirable dustiness, were simultaneously determined as previously described [93] and well suited to characterizing these CNT/F materials. Total dustiness was the percent of the total dust (sample using a closed face cassette) that can be aerosolized from the test sample, while the respirable fraction (sampled with a cyclone) was the percent of the aerosolized dust that can penetrate to the deep airways, or the alveolar region. Total dustiness may be approximated to the inhalable dustiness fraction, particularly with these CNT/F materials [94] MW #1, #2, #5, and CNF #1 had total dustiness that ranged from 3 to 14% and a respirable dustiness that ranged from 0.8–2.4%. CNF #2 was not measured but was expected to be very close to the values of CNF #1 and MW #5 published previously [93]. The total dustiness of MW #3, #4, #6, and #7 ranged from 0.2–0.5%, approximately an order of magnitude less that the other materials. The respirable dustiness ranged from 0.08–0.20%. The results indicate greater dustiness for some CNT/F compared to others but not a consistent pattern with relationship to physical dimensions or surface area.

### Density

As CNT/F mostly occur as agglomerates, the aerodynamic behavior is determined by the effective density of the agglomerates [5]. Most CNT/F exposures are performed on a mass basis and the NIOSH REL is based on mass concentration of elemental carbon. Given that density is directly proportional to mass, theoretically, the lower the effective density, the more CNT/F particle would be needed for equivalent dosing by mass. Recent computational modeling of engineered nanomaterials included density in the analyses [95–97] with some indication it was a primary driver of toxicity [96]. Measurements of bulk and tapped skeletal density were performed for all CNT/F (Table 3). MW #1, #3, and #4 were comparatively denser than MW #2 and MW #5 by an order of magnitude. The remaining materials, MW #6, MW #7, CNF #1, and CNF #2 were intermediate from the above-mentioned materials. As concluded with dustiness, there was no apparent consistent pattern that linked skeletal density to other physical dimensions, surface area, or dustiness.

### Chemical and metal impurities

Chemical and metal impurities from the catalysts and production process were usually present at some level in CNT/F end products. Some common metal impurities found in the CNT/F include iron, nickel, chromium, cobalt, copper, zinc, molybdenum and aluminum. Some of these metals such as iron [98], nickel [99], molybdenum [100], chromium and cobalt [50] were found to influence the toxicological profile of CNT. Thirty-one metals and chemical impurities were screened using inductively coupled plasma atomic emission spectroscopy (ICP-AES). Most of the thirty-one metals evaluated were below their respective analytical limits of detection (LODs). The metals that were present in one or more CNT/F include iron (0.27–6.2%) and aluminum (0–2.2%) (Table 3). Trace amounts of cobalt, molybdenum, zinc, nickel, manganese, lead and cadmium were in range of (0–0.17%), (0–0.05%), (0–0.1%), (0–0.004%), (0–0.006%), (0–0.002%) and (0–0.0005%) respectively. Most of the CNT/F currently used in U.S. facilities had minimum trace amounts of metal residues. Iron was a consistent catalyst ranging from 0.27 to 6.17% (Table 3). MW #6 and #7 had the highest levels of residual iron catalyst. Another metal of note was aluminum which was present in MW #3 at 2.1% with residual amounts of 0.31% or less in other CNT/F (Table 3). All other metals were at levels of 0.17% or less.

### Thermal stability, degradation and purity

Thermal stability, degradation, and purity of CNT/F was assessed using thermogravimetric analysis (TGA). This technique analyzes change in the weight of a specimen in relation to increasing temperature. The oxidation onset temperature, the temperature at which the oxidation of CNT/F starts, is considered a measure of thermal stability and degradation varied across the CNT/F. The onset temperature for the CNT/F ranged from 550 to 735 °C (Table 3). The residual ash, or the content left after complete oxidation, was evaluated to determine the purity of the CNT/F. The percentage of residual ash for MW #1–7 and CNF 1–2 was 1.74 ± 0.01% (means ± SD), 3.98 ± 0.26%, 8.21 ± 0.26%, 4.75 ± 0.07%, 1.11 ± 0.28%, 7.88 ± 0.15%, 8.95 ± 0.29%, 1.79 ± 0.12%, and 2.21 ± 0.16% respectively (Table 3). These values are primarily indicative of metal content and were generally consistent with relative levels of residual metal catalysts determined using ICP-AES. MW #3, #6, and #7 had the greatest residual ash and results are consistent with the higher amount of metal catalyst measured.

### Polycyclic aromatic hydrocarbons and endotoxin

Airborne background contaminants and byproducts like polycyclic aromatic hydrocarbons (PAHs) and endotoxin, a component of the bacterial cell wall, can be a major influence on the toxicity profile of various engineered nanomaterial and environmental particulates [101–103]. Previous exposure and emission monitoring at a CNF production facilities indicated the presence of PAHs with an average concentration up to 336 μg/m^3^ [104]. To rule out the influence of PAHs and endotoxin, gas chromatography–mass spectrometry with selected ion monitoring (GC–MS SIM) and limulus amebocyte lysate assay were performed, respectively. The levels of PAHs and endotoxin in the CNT/F were below their LODs. The lack of endotoxin was supported by no significant induction of tumor necrosis factor-α production (described below) from epithelial cells at the highest CNT/F dose tested.

### Acellular reactivity

Multiple physicochemical characteristics of the CNT/F including residual metal catalysts, surface defects, functionalization, and redox active organic matter, such as quinones, will alter the reactivity of the nanomaterial in biological matrices. This can lead to an imbalance in redox homeostasis that can trigger oxidative stress and toxicity. The ferric reducing ability of serum (FRAS) assay was used as an acellular screen to determine the antioxidant capacity, or the ability of CNT/F to react in biological matrices and deplete antioxidants. This assay serves as a screen for oxidative stress and potential toxicity [105]. Compared to untreated serum, reaction with CNT/F reduced the antioxidative capacity of serum by 65–100% (Table 3). CNF #1 and 2 had 100% remaining antioxidative capacity, indicating that these materials were the lowest in their ability to independently react and induce oxidative stress. MW #1 and MW #7, two very distinct CNT materials in terms of physical dimensions, consumed among the most serum antioxidants as indicated by the low remaining % antioxidative capacity. The remaining CNT ranged from 75 to 91%.

### Grouping CNT/F by principal component analysis of physicochemical characteristics

**Fig. 6:**
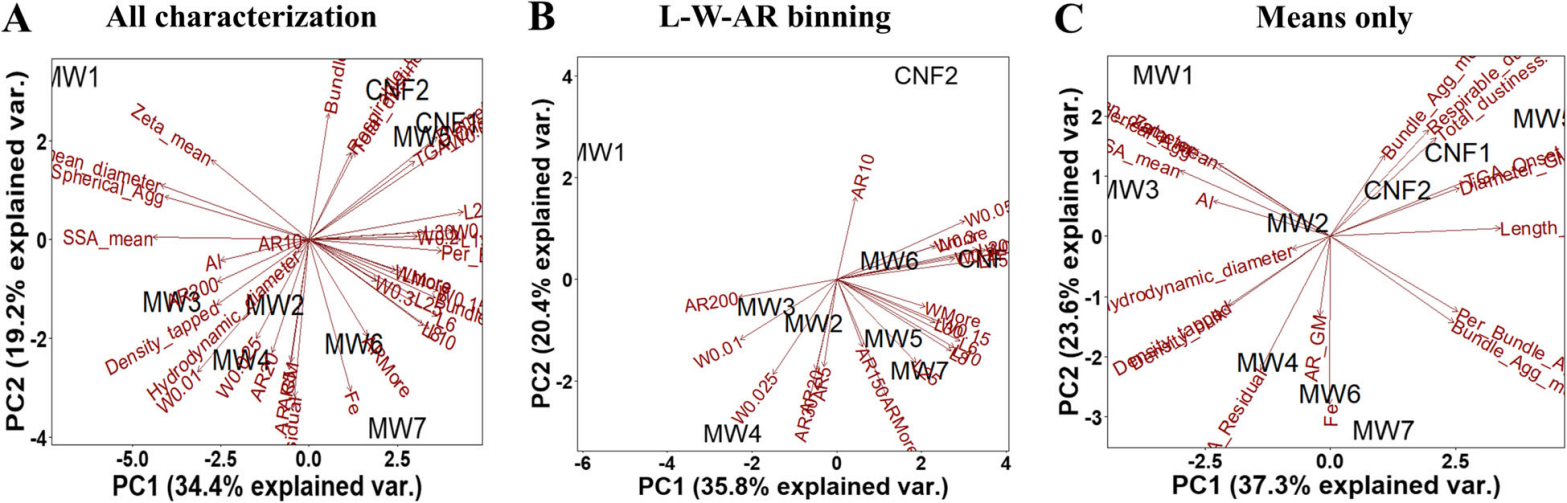
Principal component analysis (PCA) of different CNT/F materials comparing ‘all characterization’ parameters (**a**), length – diameter – aspect ratio physical dimension (**b**; L-W-AR binning) and means only (**c**) physicochemical characteristics. The first two principal components (PC), PC1 and PC2, define the x- and y-axes of the scatter plots, respectively. The distance between two materials reflects the proximity in physicochemical properties between them. PC1, PC2 and PC3 together accounted for ~ 71, 68 and 82% of the contribution to the variance in the case of all characterization, L-W-AR binning and means only, respectively. The scatter plot of the PCA along with vectors depicting the loadings of variables is shown

As a first step, feature selection using the Boruta algorithm was performed on three sets of physicochemical property data for the nine different CNT/F materials: 1) detailed characterization of length (L), diameter/width (labeled as W for figure clarity for easier distinction from L), and aspect ratio (AR) from the binned data from Figs. 3, 4, and 5 (Fig. 6b; L-W-AR binning); 2) standard physicochemical data using means only from Tables 1, 2, and 3 (Fig. 6c; Means only); and 3) the combination of L-W-AR and means only data (Fig. 6a; All characterization). Figure 6 displays the principal components analysis (PCA) results for different CNT/F samples with confirmed variables of importance from the three separate analyses (Supplemental Fig. S1 A-C). It should be noted that the PCA plots did not change without feature selection (Supplemental Fig. S2). The first three principal components describe ~ 71, 68 and 82% of the total variability among materials for the ‘all characterization’, ‘L-W-AR binning’, and ‘means only’ parameters, respectively. Most importantly, the PCA analysis of L-W-AR and all characterization variables suggested a segregation of MW #1–4 materials from MW #5–7 and CNF #1–2 (Fig. 6a-b). Overall, a combination of larger lengths and widths separated one group of materials (MW #5–7, CNF #1–2) from the second group of materials (MW #1–4) (Fig. 6a-b; Supplemental Fig. S1 A-C). The categorization of MW #5–7 and CNF #1–2 together in the same group indicates common physicochemical characteristics of these materials. However, this was not the case with PCA using traditional variable data which were based on mean values only (Fig. 6c). Often, the literature reports only mean values without including the detailed size distributions for physical dimensions. Previous studies proposed that providing distributions of dimensional characteristics would better segregate different CNT/F for grouping and toxicity [50]. The difference in the material segregation between means only compared to L-W-AR binning and all characterization suggests that varying the input parameters will influence conclusions drawn in terms of which physicochemical characteristics may drive specific toxicity outcomes. The variance in the PCA plots provided two initial suggestions: 1) binning of the physical dimensions may be critical for accurate representation of the materials and potential toxicity and 2) binning of the physical dimensions without significant additional physicochemical characterization may alone be enough to group CNT/F. The latter point agrees with the lack of a consistent pattern when comparing surface area, density, residual metal catalyst, dustiness, etc. for the various CNT/F.

Another important finding was that the use of L-W-AR binning alone parameters further separated CNF #1 from CNF #2 and grouped CNF #1 together with MW #5–7 (Fig. 6b). Furthermore, a close-clustering of CNF #1 with MW #6 and their overall grouping with MW #5 and #7 along with correlated L-W-AR binning variables in the PC1 dimension, supports the notion that a greater range of sizes can be found in CNF #1 compared to CNF #2. The PC1 dimension correlates MW #6 and CNF #1 materials with L15, L10, Lmore, W0.1, W0.2 and W0.3 variables. Similarly, a correlation of MW #5 and MW #7 with the variables L6, L8, L10, L25, L30, W0.15 and Wmore was also observed. Overall, these results suggest that larger lengths and diameters separate MW #5–7 and CNF #1 from the rest of the materials investigated. Importantly, the separation does not indicate a large fraction of the CNT/F sample has those larger dimensions (Fig. 3 and 4) but rather the sample contains some proportion of tubes with those specific nominal physical dimensions.

### *In vitro* toxicity assessment

#### Cell viability

**Fig. 7:**
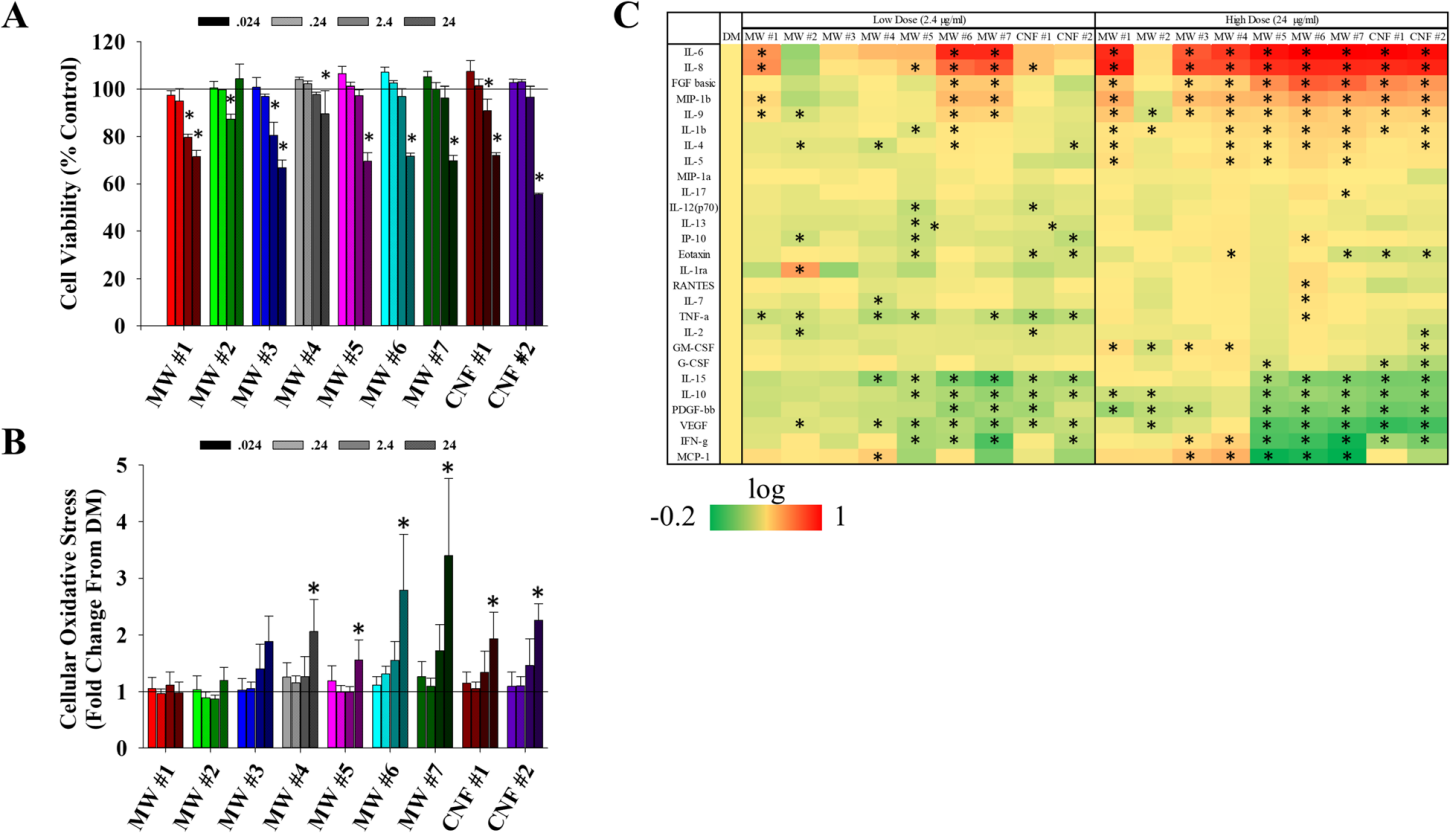
Toxicity assessment of BEAS-2B cells exposed to CNT/F. **a** WST-1 cell proliferation assay was used to assess the viability of BEAS-2B cells following exposure to increasing concentrations (0.024–24 μg/ml) of CNT/F. The dose at which the particle significantly reduced cell viability is indicated with an asterisk (*p* < 0.05). **b** Oxidative stress was measured using the CellROX assay. * p < 0.05 fold change vs. control cells represented as a reference line. **c** Protein secretions from cells exposed to 2.4 or 24 μg/ml of various CNT/F for 24 h represented as heat maps of fold change from controls with no exposure.. Significant changes from control cells were indicated with an asterisk (* p < 0.05). Log fold change was represented by color with green indicating a decrease in protein concentration and red indicating an increase on a scale of − 0.2 to 1. (*p < 0.05)

Human bronchiolar epithelial cells (BEAS-2B; selection detailed in Methods) were challenged with the nine CNT/F at 0.024, 0.24, 2.4, and 24 μg/ml for 24 h and cell viability was assessed by measuring the reduction of cell proliferation reagent WST-1 (Fig. 7a). Dose selection and relevance is detailed in the Methods. The lowest two doses (0.024 and 0.24 μg/ml) caused no significant change in cell viability. The highest dose (24 μg/ml) significantly reduced viability with all the materials tested except with MW #2. CNF #2 induced ~ 45% reduction in cell viability. The 2.4 μg/ml dose induced a small but significant reduction in cell viability for MW #1–3 and CNF #1. These toxicity results are consistent with previous results [106–108]. The IC_80_ for MW #1–7 and CNF #1–2 ranged from 11 to 43 μg/ml. Subsequent studies of genotoxicity were done at 0.024 and 2.4 μg/ml in accordance with OECD TG487 [109] and ICH S2(R1) [110] guidance for 80% or greater cell viability.

#### Oxidative stress

Reactive oxygen species (ROS) consisting of hydrogen peroxide, singlet oxygen, superoxide anion, hydroxyl radical, and hypochlorous acid are constantly regulated by the cells, which is essential to maintain homeostasis. Epidemiology studies of workers exposed to CNT/F during their manufacturing or use in downstream applications found alterations in oxidative stress markers and antioxidant enzymes [20, 23, 28, 100]. Animal and *in vitro* studies using various cell types, including epithelial cells, confirmed induction of oxidative stress with various CNT/F exposures. The response was amplified by metal impurities and was found to be dependent on the physicochemical characteristics that influence the reactivity, cellular internalization, and biopersistence [67, 111, 112]. In order to assess the oxidative stress potential of the nine CNT/F, BEAS-2B cells were exposed for 24 h at concentrations of 0–24 μg/ml and then labeled with CellROX, a non-fluorescent cell-permeant dye that fluoresces upon oxidation by ROS. Fluorescence per cell was evaluated by flow cytometry. Only the highest dose (24 μg/ml) induced a significant oxidative stress response for MW #4–7, and CNF #1–2 (Fig. 7b). There was a trend for an effect in MW #6–7 and CNF #1–2 at 2.4 μg/ml. The CNT/F with smaller physical dimensions (MW #1–3) did not induce ROS even at the highest concentration tested.

#### Cytokines, chemokines, and growth factors

A selection of 27 cytokines, chemokines, and growth factors were assessed from cell supernatant following exposure to 2.4 and 24 μg/ml of each of the nine CNT/F for 24 h (Fig. 7c). Many of the measured proteins were altered for most of the CNT/F tested. MW #2 exposure altered the least number of proteins. The reduced response was likely due to the large bundled aggregate fraction (Table 2) not having the same cellular effect as the other CNT/F. MW #6 and #7 caused the most significant changes, especially at the lower dose evaluated, indicating these materials may be more adept at altering cellular signaling than other materials in this study. All materials except MW #2 induced a significant increase in primary modulators of innate inflammation, IL-6, IL-8, IL-1β, etc., at the high dose and several at the low dose (e.g., MW #6–7). Some molecules assessed, including IL-10, an anti-inflammatory cytokine, were significantly reduced. FGF was increased while other growth factors measured, VEGF and PDGF-ββ, were generally decreased. At the higher dose, suppression of certain cytokines was more evident with MW #5–7 and CNF #1–2.

#### Genotoxicity

The potential for CNT/F to cause carcinogenicity is an area of active research [113, 114]. *In vivo* and significant *in vitro* evidence suggested adverse health consequences following inhalation to CNT/F. One material, MWCNT-7/Mistui-7, has been shown to be a complete carcinogen in rodent models, which led IARC to designate this material as possibly carcinogenic to humans (Group 2B) [34]. All other materials were considered as Group 3 as there was insufficient evidence to classify otherwise [66]. The 2020–2024 Report of the Advisory Group to Recommended Priorities for the IARC Monographs indicates MWCNT as a high priority and ready for evaluation within five years [35]. While human health effects studies have begun globally, the latency for carcinogenicity has not been reached [20–28].

**Fig. 8:**
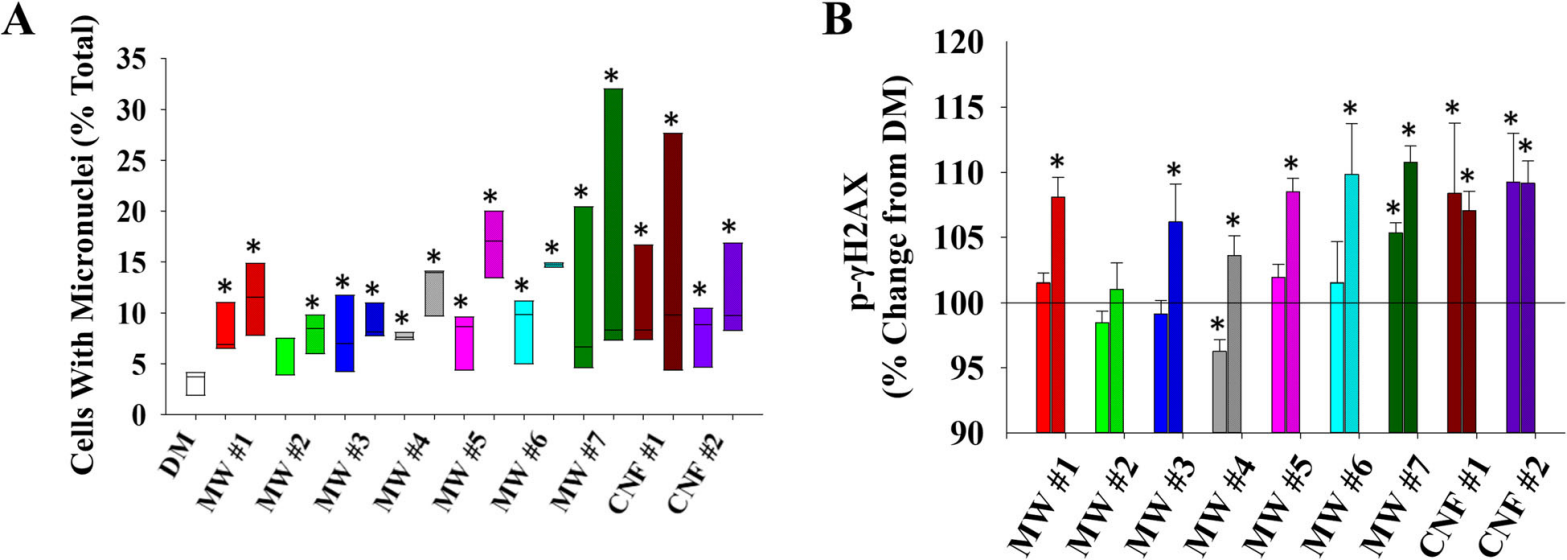
Genotoxicity assessments. **a** Cells with micronuclei were quantified and presented as percentage of total cells at treatments of 0.024 and 2.4 μg/ml. **b** Inference into double stranded DNA breaks were quantified via detection of γH2AX. Percentage change from DM is presented on the y-axis. *p < 0.05 represents significant change from control

To date, most studies concerning the potential carcinogenicity of CNT/F have used *in vitro* approaches to evaluate genotoxicity. The approach allows for a rapid screening after which detailed mechanistic and *in vivo* studies can be conducted to expand initial evidence of genotoxicity. The micronucleus assay was used to determine if CNT/F treatment results in disruption of the mitotic spindle or chromosome breakage. This approach also allows for the simultaneous evaluation of a large group of materials. Parallel cultures of human epithelial BEAS-2B cells were exposed to 0.024 and 2.4 μg/ml of the 9 CNT/F, with MW#5 (Mitsui-7/MWCNT-7) serving as a documented positive control, for 24 h and the number of cells with micronuclei were quantified (Fig. 8a-b). The screening approach, including cell type and exposure concentration, has been used previously by our group [10, 106, 108]. Viability in the high dose was ≥80%, and the low dose had ≥97% viability. In DM-exposed cells, few micronuclei were detected, and background incidence was similar to previous studies [10, 108]. All CNT/F materials at both the low and high dose induced significant increases in micronuclei number except for the low dose of MW #2 (Fig. 8b). The treatments were not significantly different from one another.

In complement, the phosphorylation of H2AX, a cellular response to repair double-strand DNA breaks, was evaluated. Flow cytometry was used to quantify phosphorylated H2AX, or γ-H2AX. All high dose-treated cells induced γ-H2AX except for MW #2 (Fig. 8c). Increased levels of γ-H2AX were also measured for MW #7, CNF #1 and CNF #2 for the low dose treatment. For CNF, the low and high doses had similar effects. While measurements of γ-H2AX was considered a low priority indicator of genotoxicity as it does not directly indicate irreversible mutations [114], the response was similar to the micronuclei outcome.

### Hierarchical clustering and PCA of the cellular outcomes

**Fig. 9:**
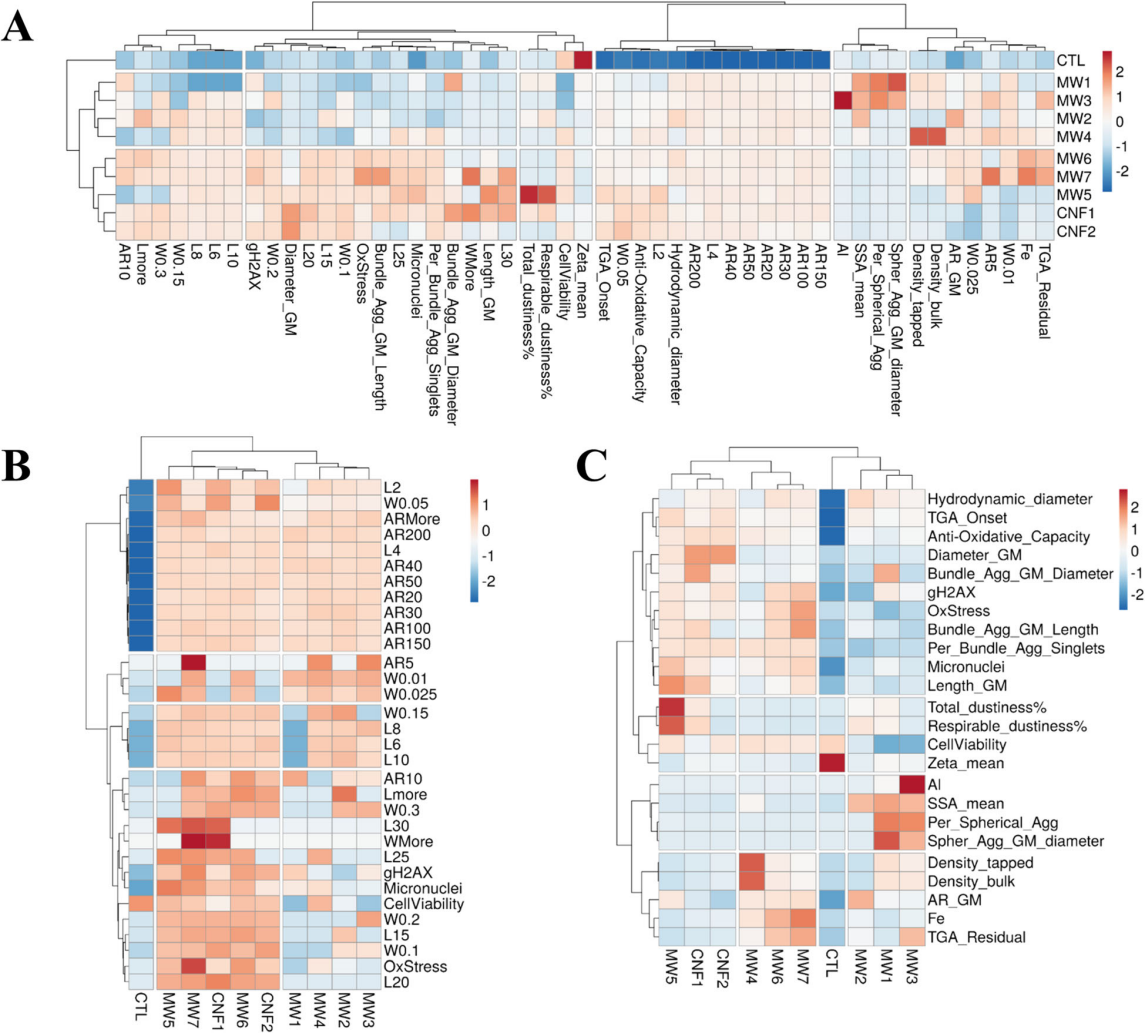
Clustering of physicochemical characteristics with the epithelial cell toxicity outcomes of cell viability, cellular oxidative stress, γH2AX and micronuclei formation. Toxicity outcomes compared to (**a**) all characteristics, (**b**) L-W-AR binning, and (**c**) means only were presented

A hierarchical clustering analysis (HCA) was performed to distinguish or discriminate the BEAS-2B cellular outcomes induced by CNT/F materials with varying physicochemical characteristics. HCA, unlike model-dependent analyses such as supervised machine learning methods, is a model-free statistical approach that makes no a priori assumptions about the class identification of data. The resulting dendrogram from the HCA analysis of physicochemical properties of ‘all characteristics’ combined with outcomes of the four primary *in vitro* assays, cell viability, cellular oxidative stress, micronuclei formation, and γ-H2AX, is depicted in Fig. 9a. Overall, the dendrogram initially divided CNT/F exposure responses into two clusters or groups, one predominantly containing MW #1–4 together with the control group, and the other containing MW #5–7 and CNF #1–2 (Fig. 9a). HCA was also done for outcomes in comparison to the ‘L-W-AR binning’ and ‘means only’ characterization profiles. The L-W-AR binning profile produced the same two clusters (Fig. 9b) as developed using all characterization parameters (Fig. 9a). The means only HCA shifted MW #4 into the cluster with MW #5–7 and CNF #1–2, suggesting similarities resembling more MW #6–7 than MW #1–3 (Fig. 9c), indicating the input selection of characteristics can vary the grouping in relation to toxicity outcomes. PCA results from ‘all characterization’ combined with the four primary *in vitro* assay outcomes (Supplemental Fig. 3A) grouped similarly to ‘L-W-AR binning’ (Supplemental Fig. 3B), producing a separation between the two clusters. The ‘means only’ with *in vitro* outcomes (Supplemental Fig. 3C), like the HCA dendrogram (Fig. 9c), was less clear in distinguishing groups of CNT/F.

HCA analysis was done for ‘all characteristics’ and the four primary *in vitro* outcomes along with inflammatory protein production (Supplemental Fig. 4). The grouping was unaltered compared to Fig. 9a except for a clearer separation in the two sub-clusters. This was evident as MW #6–7 had a significant grouping of induced inflammatory proteins compared to MW #5 and CNF #1–2. What became evident from Fig. 9a-b and Supplemental Fig. 4 was that binning of the aspect ratio data did not segregate to any particular outcome and may be unnecessary for the HCA. To illustrate, Supplemental Fig. 5, HCA without aspect ratio binning, created the same two clusters of MW #1–4 and MW #5–7 / CNF #1–2 for ‘all characterization’ (Supplemental Fig. 5A) and ‘L-W-AR binning’ (Supplemental Fig. 5B) when considering the four primary *in vitro* epithelial toxicity outcomes. Within each sub-cluster, co-clustering between materials was also evident as all variations in HCA pulled out the two CNF from the CNT and MW #6 and #7 clustered together even without secreted protein changes as in Supplemental Fig. 4. There were a few subtle differences in pairings between MW #1–4 and control for the L-W-AR binning HCA compared to all characterization. As an additional step, HCA analysis was done, without aspect ratio, to include the altered protein changes with the four primary toxicity outcomes and the three variations in characterization parameters. Interestingly, all three scenarios (Supplemental Fig. 6A-C) now had the same two clusters, meaning the ‘means only’ HCA placed MW #4 with MW #1–3 instead of MW #5–7 and CNF #1–2. Previously for ‘mean only’ HCA (Fig. 9C), MW #4 was combined with MW #6–7. It was clear from Supplemental Fig. 6C that the large group of induced inflammatory proteins for MW #6–7, not seen with MW #4, altered the clustering. This series of analyses suggests that when using ‘means only’ for physicochemical characterization, additional toxicity data may be necessary to accurately categorize all materials in terms of epithelial cell toxicity. It also indicated that altered inflammatory protein concentrations, at least the panel used in this study, were not necessary to group CNT/F in terms of epithelial toxicity if binning of physical dimensions was available.

We next considered just the four primary outcomes of *in vitro* toxicity and protein production with no physicochemical characteristics. The HCA analysis also grouped MW #1–4 separately from MW #5–7 and CNF #1–2 (Supplemental Fig. 7). The toxicity only grouping consistently matched HCA analyses using ‘all characterization’ or more simply the ‘L-W-AR binning’ as compared to the ‘means only’ characterization from Fig. 9. The separation of oxidative stress, micronuclei formation, and γ-H2AX from protein production when considering outcomes only (Supplemental Fig. 7) further supports the consistency of grouping when physical dimension binning was determined and analyzed without inflammatory protein production (Supplemental Fig. 4 and 6). The separation also suggests that epithelial cell viability and inflammatory cytokine production, as assessed by the panel used, were not primary drivers of genotoxicity.

The various analyses allowed for interpretation of which physicochemical properties drive which epithelial cell toxicity outcomes. Three of the four *in vitro* outcomes evaluated, oxidative stress, micronuclei formation, and γ-H2AX, grouped with certain physicochemical properties that were identified in Fig. 9 and Supplemental Figs. 4, 5, and 6 as distinguishing between the two CNT/F clusters. Bins of larger lengths and diameters (W), including L15, L20, L25, L30, W0.1, W0.2, and Wmore, clustered with the outcomes. Also clustering with the toxicity outcomes were bundled agglomerate singlet percentage, length, and diameter from the two-dimensional sizing (Table 2). Inherently, that would be expected as the increasing physical dimensions of length and diameter transition the CNT/F from a spherical agglomerate (e.g., MW #1 and #3) to a more elongated bundled agglomerate. Depending on the parameters for HCA, the fourth primary outcome, cell viability, sometimes grouped with the other three toxicity variables of importance (Fig. 9b; Supplemental Fig. 4), but other times did not (Fig. 9a; Supplemental Fig. 5 and 7), suggesting that cell viability may not always be a useful assay for determining differential toxicity among materials. Overall, MW #1–4 materials clustered separately from MW #5–7 / CNF #1–2. While all materials induced significant micronuclei formation (Fig. 8b), when combined with γ-H2AX (Fig. 8c) and cellular oxidative stress (Fig. 7b), there was a propensity for greater severity in the cluster of materials that contained a greater proportion of tubes/fibers with larger physical dimensions, MW #5–7 / CNF #1–2 (Fig. 9). This was further supported by the fact that control samples always clustered with MW #1–4.

Of note was the close clustering of cellular oxidative stress with γ-H2AX levels and micronuclei formulation. This was especially evident with physicochemical variables of larger lengths and widths and bundled agglomerate characteristics. The finding was consistent of genotoxicity through indirect oxidative stress–related mechanisms upon exposure to high aspect ratio nanomaterials [115, 116]. Increased oxidative stress, as a result of lysosomal damage by inefficient phagocytosis of high aspect ratio nanomaterials, can cause double-stranded DNA damage and chromosomal aberrations leading to micronuclei formation. γ-H2AX, an early indicator of DNA-double strand breakage and a process that precedes the formation of micronuclei, segregated mostly with larger nominal tube/fiber diameter and length of CNT/F. Oxidative stress responses clustered together with bundled agglomerate length support the notion that oxidative stress due to inefficient cellular handling of aggregated particles and/or lysosomal damage by particles of larger dimensions could be a contributing mechanism of genotoxicity, especially for MW #5–7 and CNF #1–2.

Caution should be taken not to overstate the associations of the larger CNT/F as SWCNT have been shown to be potent inducers of chromosomal damage [10, 114, 117]. Also, MW #1 and #3 in this study induced significant effects complementing a recent pulmonary exposure study of a MWCNT 7.5 nm in diameter induced cancer [118]. Kuempel *et al*. concluded in a review of CNT genotoxicity studies that there was not a straightforward relationship between length and genotoxicity, although most of the evaluated studies had CNT length of only a few microns or less [113]. Jackson *et al*. (2015) and Poulsen *et al*. (2016) described increased diameter as a physicochemical characteristic linking genotoxicity for the 15 and 10 MWCNT tested in those respective studies [41, 45]. Those studies represented materials similar to MW # 1–5. The greater range of CNT/F physical dimensions in this study provided a clearer separation of materials. Overall, all materials, from MW #1 to CNF #2, had the potential to induce *in vitro* genotoxicity. When combining cellular oxidative stress and γ-H2AX with micronuclei formation and a broad sampling of the class of CNT/F there was a general shift for greater length and diameter materials to cluster together with some increased severity.

Many of the other physicochemical characteristics did not associate with epithelial cell toxicity outcomes. This does not indicate a lack of importance but more the scope of the endpoints considered. Subsequent studies will evaluate the association of the various physicochemical characteristics with macrophage activation, fibrosis development, and translocation. It was noted that while cellular oxidative stress consistently clustered with micronuclei formation and γ-H2AX, there was no clustering with acellular reactivity measured by FRAS or residual metal catalyst. This indicates the residual metal catalyst levels ranging from 0.3–6% were not the primary drivers of cellular oxidative stress compared with larger physical dimensions. The clustering was confirmed by residual metal catalyst grouping with residual ash measured by TGA as expected (Fig. 9; Supplemental Fig. 4). In conjunction with anti-oxidative capacity (acellular oxidative stress), aspect ratio, hydrodynamic diameter, and the smaller length bins (L2 and L4) were unable to segregate materials and clustered away from all toxicity outcomes. Additionally, density, specific surface area, zeta potential, the smaller width bins, and spherical agglomerate measurements were also not predictive. Dustiness, while a critical factor for worker exposure assessment, was not predictive of epithelial cell toxicity. Cluster analysis of all the induced cytokines together with physicochemical characteristics and toxicity outcomes measured (Supplemental Fig. 4) placed the proteins into four groups, two of which were exclusive for proteins. Most proteins did not cluster with the physicochemical characteristics and none with the four primary biological outcomes. While significant changes in inflammatory drivers, growth factors, and cell survival and proliferation signaling molecules occur following CNT/F treatment in the epithelial cells, the change in these proteins did not correlate to biological outcomes like oxidative stress and genotoxicity.

### Summary

Advances in computational analysis are being applied to the almost two decades of engineered nanomaterial research for grouping and understanding the physicochemical drivers of toxicity [95–97, 119, 120], including studies of carbon nanotubes [45, 50, 77, 121, 122]. The analyses of the data from this study illustrate that detailed physical dimension characteristics provide a more consistent grouping of CNT/F as compared to using only data means. In fact, analysis of binning of nominal tube physical dimensions alone produced a similar grouping as to all characterization parameters. Theoretically, working backwards, a predictive algorithm could be generated that allows classification of CNT/F into distinct toxicity groups based on 200 paired length and diameter measurements. While all materials induced micronuclei formation in human bronchial epithelial cells, when combined with additional parameters associated with genotoxicity, there was an increase in the severity if the sample contained some proportion of materials with larger diameters and longer nominal lengths. The population of nominal tubes with longer length and larger diameters within a sample was not always the majority (e.g., MW #7), meaning a significant percentage of the tubes with those characteristics was not needed for increased severity of toxicity. The analyses indicate that a more detailed physicochemical characterization of physical dimensions provides better understanding of the differential toxicity within a class of materials, implying that evaluating particle characteristic means alone may not be sufficient to accurately segregate CNT/F for certain aspects of toxicity. Subsequent studies analyzing outcomes of inflammation, histopathology, and translocation following CNT/F exposure will further develop clustering by physicochemical characteristics and specific endpoint toxicity. In this study evaluating epithelial cell toxicity, all materials induced some level of genotoxicity. However, of the CNT/F evaluated, materials that contained a proportion of tubes with greater lengths and diameters were associated with increased severity.

General Conclusions:

- Binning of physical dimensions (length and diameter/width) offered greater resolution in terms of grouping CNT/F based on physicochemical characteristics compared to using means only. This was further evident when analyzing the physicochemical characteristics and epithelial cell toxicity outcomes.
- Binning of physical dimensions alone offered the same resolution for grouping CNT/F as using all physicochemical characteristics suggesting the potential of reduced characterization needed for grouping CNT/F fibers.
- All CNT/F, with the lone exception of the highly aggregated low dose of MW #2, induced genotoxicity. There was no difference between materials for micronuclei formation.
- When micronuclei formation was combined with cellular oxidative stress and γ-H2AX levels, CNT/F with increasing length and diameter grouped with slightly more toxicity.
- Computational analysis illustrated that increasing length and diameter contribute to greater epithelial cell toxicity. Binning of physical dimensions alone was sufficient to group CNT/F in terms of epithelial cell toxicity. The nature of the bundled agglomerate formation, a reflection of the physical dimensions, also grouped with toxicity outcomes.
- The increasing length and diameter CNT/F do not need to be the majority constituent of the produced material. A small percentage of nominal tubes/fibers with increased length and diameter was sufficient to alter the toxicity profile.
- There was no consistent pattern of density, specific surface area, dustiness, residual metal catalyst, and surface charge associating with physical dimensions or genotoxicity outcomes.

## Methods

### *Materials*

All CNT/F used in this study, with the exception of MW #5 (MWCNT-7/Mitsui-7/), were as-produced materials obtained from six different U. S. primary or secondary manufacturing facilities. Occupational exposure assessments of these facilities were completed to provide insight into human exposure risks and offer direct insight into the vast array of materials utilized [29]. MW #5 (MWCNT-7/Mitsui-7/) was included in this study as a benchmark material as its toxicity profile is well-studied and characterized (Fig. 1).

### Characterization

#### Length and diameter

Tube and fiber length and diameter were measured using high resolution scanning transmission electron microscopy as previously described [32]. Briefly, samples of CNT/F were sonicated in isopropanol for 5 min. A STEM grid was dipped into the dispersed suspension and used for imaging and analysis. Measuring tools included in the electron microscope’s software were used to determine paired length and diameter. Length was determined by connected points at the two extremes without following the curvature of the nanotube or nanofiber. High resolution images were collected with a Hitachi HD-2300 STEM.

#### Two-dimensional agglomerate sizing

Samples of CNT/F were prepared in physiologic dispersion medium and the dispersed samples were prepared for field emission scanning electron microscopy (FE-SEM; Hitachi S-4800, Tokyo, Japan). Measurements were collected using measuring tools of the microscope’s provided software (FE-PC SEM Ver. 2.8, Hitachi High Technologies America). The largest crosswise diameter of 75 agglomerates were measured for each material. Materials were subsequently categorized into distinct groups of agglomeration defined as either spherical agglomerates or bundles of fibers with one dimension greater than three times the other dimension, referred to as bundled agglomerates. Bundled agglomerates had both a diameter and length measurement.

#### Aspect ratio

Aspect ratio was calculated as the ratio of CNT/F length to diameter.

#### Hydrodynamic diameter

The hydrodynamic agglomerated size of the various CNT/F dispersed in DM was evaluated using DLS. DLS was performed on a Malvern Zetasizer Nano ZS90 (Worcestershire, UK) equipped with a 633 nm laser at a 90^o^ scattering angle. The DLS measurements were performed by dispersing the CNT/F material in dispersion media. After two minutes of equilibration inside the equipment, five measurements, each consisting of at least five runs were recorded.

#### Surface area

Using Brunauer Emmet Teller (BET) methodology, the surface area of each CNT/F was measured as described previously [89]. Briefly, samples were degassed in ultrahigh purity (UHP) nitrogen for 30 min at 90 °C, and then for 90 min at 200 °C. The surface areas were determined by a 5-point BET measurement with UHP nitrogen as the adsorbate and liquid nitrogen as the cryogen.

#### Zeta potential

Zeta potentials were measured using a Nano ZS90 instrument (Malvern Instruments, UK). Viscosity of the control medium was previously determined at room temperature using a VS-10 viscometer (Malvern Instruments) and used as the value for calculation of zeta potential. The pH of all samples was measured using a calibrated electrode.

#### Dustiness

Dustiness is a unitless measurement (mass/mass) measured using the Venturi dustiness device as was previously described [93]. This measurement represents a percentage of total (~inhalable) and respirable airborne mass normalized to the quantity of test powder prior to dispersion.

#### Density

Skeletal density of each CNT/F was determined based on ISO 23145. For tapped density, a 10 ml graduated cylinder was tared on a calibrated analytical balance and the material was added. To measure tapped density, the container was gently tapped, and the level of the powder was recorded to the nearest 0.1 ml. The cylinder with powder was reweighed. Density was calculated as the mass of powder divided by volume. The measurement was replicated three times for each sample and the results are expressed as means ± standard deviation.

#### Metal analysis

ICP-AES was used to measure metal content. Digestion was completed using a microwave digestion system (MARS, CEM). Five mg of each sample and 10 ml of concentrated nitric acid were added to the digestion vessel and were subsequently digested using the following program: maximum power 400, 100% power, ramp 20 °C/min, 600 psi, temperature 230 °C, hold time 60 min. Samples were then heated on a hot block to reduce the volume to 1 ml. The samples were then brought to a volume of 10 ml using deionized water. Sample digests were analyzed according to NMAM 7300. Metal analysis included aluminum, antimony, arsenic, barium, beryllium, cadmium, calcium, chromium, cobalt, copper, iron, lanthanum, lead, lithium, magnesium, manganese, molybdenum, nickel, phosphorus, potassium, selenium, silver, strontium, tellurium, thallium, tin, titanium, vanadium, yttrium, zinc, and zirconium.

#### Endotoxin

Endotoxin contamination was measured using the Limulus amebocyte lysate test according to the manufacturer’s protocol. The limit of detection was 0.1 EU/ml.

#### PAH

PAH levels were quantified by gas chromatography–mass spectrometry with selected ion monitoring (GC–MS SIM) using method previously described [104]. Briefly, dry samples of CNT/F were extracted in 10 ml methylene chloride with shaking for two minutes. The samples were extracted three times and the extracts were combined. The Limit of Quantification (LOQ) and Limit of Detection (LOD) and other details were provided previously [104].

#### TGA

Thermogravimetric analysis (TGA) was performed to determine the residual ash contents and thermal stability of the materials. Samples were analyzed as previously described using a Q50000IR TGA (TA Instruments Inc., New Castle, DE) [89].

#### Acellular oxidative potential

The acellular oxidative potential of CNT/F was determined using ferric reducing ability of serum (FRAS). Serum is a complex mixture consisting of various forms of antioxidants that can quench chemically distinct oxidants. The oxidative potential of the CNT/F was determined by reacting human blood serum (HBS; Sigma-Aldrich, St. Louis, MO; Cat # P2918) with CNT/F and evaluating the decrease in antioxidants in HBS. The reduction in antioxidant capacity of the serum was quantified by ferric to ferrous ion reduction and formation of a colored ferrous-tripyridyltriazine complex. The decrease in antioxidative capacity in HBS was compared with Trolox, a vitamin E analog. This modified total antioxidant capacity approach has been used to evaluate the oxidative potential of various engineered nanomaterials [123–125].

Human blood serum was rapidly thawed and exposed to CNT/F at a concentration of 5 mg/ml in low protein retention tubes. To properly disperse the nanomaterials, the samples were sonicated for 5 min. The dispersed samples were then incubated in the dark at 37 °C for three hours on an orbital shaker set at 450 RPM. The CNT/F were removed from serum by centrifuging the mixture at 14,000 g for three hours. 50 μl of the serum supernatant was reacted with 1 ml of the FRAS solution to quantify the level of antioxidant depletion. The FRAS solution is a volume mixture of 10:1:1 consisting of 0.2021 g of sodium acetic trihydrate and 1.060 ml of glacial acetic acid (Alfa Aesar, Haverhill, MA; Cat # 36289) in 100 ml of deionized water, 0.0946 g of TPTZ (2,4,6-tri(2-pyridyl)-s-triazine)(Sigma-Aldrich, Cat # T1253) and 1.2 ml of 1 M HCl in 30 ml of deionized water and 0.1635 g of FeCl_3_·6H_2_O (Sigma-Aldrich, Cat # 44944) in 30 ml of deionized water respectively. For quantitative comparison of the level of antioxidant depletion, Trolox (Sigma-Aldrich, Cat # 238813) standards were prepared at concentrations of 25–800 μM and reacted with the FRAS solution. The change in color was quantified by reading the absorption at 586 nm.

### *In vitro* study design

The goal of this study was to investigate the toxic effects of CNT/F on pulmonary epithelial cells. Immortalized human bronchial epithelial cells (BEAS-2B), cells were exposed to several concentrations of each of the nine materials. Changes in cell viability, oxidative stress, and protein production were determined. Additionally, the genotoxicity of these materials was assessed using γH2AX detection and micronuclei formation. The BEAS-2B cell line was selected as a non-tumorigenic cell line originally derived from human bronchial epithelial cells immortalized by viral transfection [126]. Since their original description, monocultures of these cells have been widely used and accepted by researchers to study genotoxicity and potential lung carcinogenesis of test agents. The BEAS-2B cells have several advantages that have made them suitable cell population for genotoxicity analysis. The cells have a stable karyotype and a low background frequency of micronuclei at early passage [127–129] [117, 32] [107, 108]. These cellular characteristics of the BEAS-2B are in accordance with the OECD guidelines as follows: “Because the background frequency of micronuclei will influence the sensitivity of the assay, it is recommended that cell types with a stable and defined background frequency of micronucleus formation and a stable karyotype be used.” Previous investigations have demonstrated that the BEAS-2B cells double every 18 to 20 h when seeded at 70% density [107, 108] [117, 128].

#### Correspondence to human exposure

The experiments were performed on μg/ml basis. As the surface area and volume required changes with the cell culture consumable used for the assays, in order to be open and enable future comparative and meta-analysis of the data generated, we have reported the concentrations in μg/cm^2^ basis alongside the μg/ml. Cellular toxicity and oxidative stress were performed at a range of approximately 0–15 μg/cm^2^ (0–24 μg/ml). Micronuclei formation and γ-H2AX were evaluated at 0.009 and 0.9 μg/cm^2^, very much at the lower end of the toxicity range.

Based on Erdely *et. al* 2013 [130], an inhalable elemental carbon mass concentration arithmetic mean of 10.6 μg/m^3^ (geometric mean 4.21 μg/m^3^) was found among workers exposed to MWCNT. The concentration equates to a deposited dose of approximately 4.07 μg /d in a human. The *in vitro* exposure of 0.009 μg/cm^2^, based on an human alveolar surface area of 102 m^2^ (1.02 × 10^6^ cm^2^) [131] corresponds to 9180 μg deposited in the human. With estimated 4.07 μg/d deposited, this would be equivalent to exposure of approximately 2250 days. Assuming 5 days/week of work the 2250 days corresponds to ~ 9 years of exposure. The *in vitro* exposure of 0.9 μg/cm^2^ corresponds to 918,000 μg deposited in the human. With estimated 4.07 μg/d deposited, this would be equivalent to exposure of 225,000 days. Assuming 5 days/week of work the 225,000 days corresponds to ~ 900 years of exposure.

#### CNT/F dispersion in cell culture media

Aqueous stock suspensions of CNT/F were generated by weighing the dry powder and suspending in well-characterized dispersion medium [DM; 0.6 mg/ml mouse serum albumin + 0.01 mg/ml 1,2-dipalmitoyl-sn-glycero-3-phosphotidyl (DPPC) in phosphate-buffered saline (PBS) without calcium and magnesium] [132] at 2 mg/ml concentration. The stock suspension was sonicated for 5 min at 70% amplitude using a cup horn sonicator (Sonics VibraCell VCX-750 with Cup-type Sonicator; Newton, CT) immersed in continuous flowing cold water. The samples were vortexed intermittently after every minute for 10 s. The stock solution at 2 mg/ml was dispersed in cell culture media by diluting to highest test concentration i.e. 24 μg/ml. The CNT/F containing cell culture media was then subjected to probe tip sonication (Branson Sonifer 450, continuous output) for a total of 2 min, with 10 s vertexing after every 30 s. CNT/F containing cell culture media at 0.024, 0.24 or 2.4 μg/ml were obtained by serial dilution.

#### Cell culture and cytotoxicity

Human bronchial epithelial cells (BEAS-2B) were obtained from American type culture collection (ATCC, Manassas, VA) and cultured in Dulbecco’s modified Eagle medium (DMEM) supplemented with 10% heat inactivated fetal bovine serum (R&D Systems Inc., Minneapolis, MN) and 1% penicillin Streptomycin (Invitrogen, Carlsbad, CA). Cells were cultured to 70% confluency in an incubator maintained at 37 °C and 5% CO_2_. Trypsin-EDTA (0.25%) was used to detach the cells from the culture flasks for sub-culturing. The cells between passage 4–10 were used and these cells had a doubling time of 18–20 h. For evaluating the cytotoxicity, parallel cultures of cells were seeded at 46,900 cells/cm^2^ overnight in a 96-well plate and dosed at a concentration of 0.024, .24 or 2.4 or 24 μg/ml to the CNT/F with MW #5 (Mitsui-7/MWCNT-7) serving as a positive control. In terms of surface area this corresponds to 0.015, 0.15, 1.5 and 15 μg/cm^2^. Parallel cells cultures were exposed to CNT/F for 24 h and challenged with fresh media containing 10% volume/volume WST-1 cell proliferation reagent (Sigma-Aldrich, Cat #5015944001). After 2 h of incubation the WST-1 consumption was recorded by measuring the absorbance at 450 nm subtracted with absorbance at 660 nm to account for turbidity/background. Cytotoxicity was evaluated by repeating the experiment on three separate days with each dose tested in quadruplicates each day.

#### Oxidative stress

Intracellular ROS formation after 24 h post exposure of the CNT/F was assessed using CellROX® Green (Invitrogen). Cells were seeded at 46,900 cells/cm^2^ overnight in a 24-well plate and dosed at a concentration of 0.024, 0.24, 2.4, or 24 μg/ml of one of the nine materials tested. In terms of surface area this corresponds to 0.012, 0.12, 1.2 and 12 μg/cm^2^. After 24 h of exposure to CNT/F, cells were detached using Trypsin-EDTA, and washed and incubated with 50 μM CellROX for 20 min. Cells were washed and fixed by incubating them with 10% formaldehyde in PBS. The change in CellROX fluorescence was captured using a BD LSR II flow cytometer (BD Biosciences, San Diego, CA). The cells were strained through a Flowmi™ Cell Strainer (Bel-Art Products, Inc. Wayne, NJ) to achieve uniform single cell suspensions and remove any aggregates. The mean fluorescence was determined using FlowJo (FlowJo LLC, Ashland, OR). The experiment was performed on four separate days with each dose tested in triplicates each day. At least 10,000 cells were analyzed per sample in each group.

#### Protein quantification

Alteration in the proteins released due to CNT/F exposure was quantified by measuring twenty- seven proteins in the supernatants after 24 h exposure to 0, 2.4 and 24 μg/ml of the CNT/F. In terms of surface area, this corresponds to 0, 0.75 and 7.48 μg/cm^2^. The proteins were measured using a BIO-RAD Bio plex Pro Human Cytokine Grp 1 Panel 27 plex (Bio-Rad Laboratories Inc., CA, Cat # M500KCAFOY). The 27 proteins measured include cytokine FGF basic, eotaxin, G-CSF, GM-CSF, IFN-γ, IL-1β, IL-1ra, IL-2, IL-4, IL-5, IL-6, IL-7, IL-8, IL-9, IL-10, IL-12 (p70), IL-13, IL-15, IL-17A, IP-10, MCP-1 (MCAF), MIP-1α, MIP-1β, PDGF-BB, RANTES, TNF-α and VEGF. These proteins are key cytokines, chemokines and growth factors that play an important role in inflammation. The assay sensitivities for these markers ranged from 0.1 to 33.3 pg/ml.

#### Double stranded DNA break

Phosphorylation of H2A histone family member X (H2AX) occurs during repair of DNA breakage and is considered a sensitive marker for double stranded DNA breakage. Flow cytometric evaluation of H2AX phosphorylation was performed as described earlier [133]. Cells were plated on a 12-well plate overnight and challenged with 1.5 ml of CNT/F dispersed in cell culture medium for 24 h. The cells were dosed with 0, 0.024 and 2.4 μg/ml of CNT/F. In terms of surface area, this corresponds to 0, 0.009 and 0.9 μg/cm^2^. After 24 h post exposure to CNT/F, the cells were lifted by trypsinization and fixed using 10% formaldehyde in PBS. Cells were permeabilized with 0.2% (v/v) Triton X-100 (Sigma-Aldrich) in PBS for 30 min followed by blocking of nonspecific binding by incubating them with 1% (w/v) of bovine serum albumin (Sigma-Aldrich) for 1 h. The cells were then incubated overnight with 1:50 dilution of Phospho-Histone H2A.X (Ser139) Rabbit mAb (Alexa fluor 488 conjugated) (Cell Signaling, Beverly, MA). The cells were strained through a Flowmi™ Cell Strainer (Bel-Art Products, Inc. Wayne, NJ) to achieve uniform single cell suspensions and remove any aggregates. Fluorescence from single cell suspensions was captured using a BD LSR II flow cytometer (BD Biosciences, San Diego, CA). The mean fluorescence was determined using FlowJo (FlowJo LLC, Ashland, OR). The experiment was performed in triplicates and at least 10,000 cells were analyzed per sample in each group.

#### Micronucleus assay

BEAS-2B cells (> 97% viability by trypan blue) were plated at 70% confluency on a two-well glass chamber slides (Thermo Scientific Nunc Lab-Tek, Waltham, MA; Cat# 154461) overnight and challenged with 1.5 ml of the 9 CNT/F dispersed in cell culture medium for 24 h. As outlined in previous investigations, fresh media was added with the test agent to the cultured cells to stimulate cell proliferation. The cells were monitored for cell rounding for 24 h following the addition of media to assure that mitosis had occurred. Cell rounding is an accepted measure of cell proliferation because most attached cells in culture round up when the cells enter mitosis [134]. The cells were harvested for analysis 24 h after the addition of fresh media and test agent to avoid growing the cells to confluence. The potency of the test CNT/F was compared to MW #5 (Mistui-7/MWCNT-7).

Parallel cell cultures were treated with 0, 0.024 and 2.4 μg/ml of the CNT/F, which included MW #5 (Mistui-7/MWCNT-7) as a reference material known to be genotoxic. In terms of surface area, this corresponds to 0, 0.009 and 0.9 μg/cm^2^. After 24 h post exposure to CNT/F, the slides were washed with PBS and fixed with an ice-cold mixture of 3:1 methanol and acetic acid for 30 min and then stained with DAPI (Vector, Burlingame, CA) for nuclear content. The cells were imaged using a laser scanning confocal microscope (LSM 780, CZ Microscopy, Thornwood, NY) using a 60x objective. The complete depth of the cell was captured by taking Z-Sections and the 3D images were converted to 2D using maximum intensity projection.. Photographs of a minimum of 100 cells per slide were taken and the number of micronuclei present was recorded, and the experiment was repeated in triplicate for a minimum of 300 cells per treatment group. Two independent observers that were blinded to the treatment groups recorded the number of micronuclei.

### Statistical analysis

*In vitro* assays of cytotoxicity and oxidative stress were analyzed using one-way (particle type) and two-way (particle type by dose) analyses of variance. Post hoc comparisons were evaluated with Fishers LSD test. Some variables were transformed using the natural log prior to analysis to meet the model assumptions of homogeneous variance. Significance was achieved at a *p* < 0.05. All analyses were carried out using SAS/STAT version 9.4 for Windows, and JMP statistical software version 12 (SAS, Cary NC).

### Feature selection and principal component analysis

To permit selection of the minimal number of features among all characterization and L-W-AR properties that could potentially discriminate between each material investigated, feature selection was performed with a random forest-based approach [135] using the “Boruta” algorithm [136] in the R statistical environment [137]. The Boruta algorithm adds randomness to the variables in the dataset by creating shuffled copies of all variables (“shadow features”). “Boruta” iteratively assesses if each variable has a higher Z-score than the maximum Z-score of its shadow features. At each iteration, variables with Z-scores lower than shadow features are deemed unimportant and removed subsequently by the algorithm to capture all the important, interesting features one might have in the dataset with respect to a dependent variable, in this case each material itself. Then, using traditional, L-W-AR, and combined variables retained after applying the “Boruta” algorithm, principal component analysis (PCA) was performed to identify significant patterns that explained the majority of the variations in the physicochemical properties among the different CNT/F materials investigated. PCA was performed using the prcomp command of the R statistical software (R Core Team, 2016).

## Supplementary Information

**Additional file 1.**

## Data Availability

All data from this project will be posted in the NIOSH Data and Statistics Gateway https://www.cdc.gov/niosh/data/default.html
