# Supplementary material for: Physicochemical characterization and genotoxicity of the broad class of carbon nanotubes and nanofibers used or produced in U.S. facilities
Source: Part Fibre Toxicol. 2020 Dec 7;17:62. doi: 10.1186/s12989-020-00392-w (PMC7720492; doi:10.1186/s12989-020-00392-w)

**Supplemental**

**Physicochemical characterization and genotoxicity of the broad class of carbon nanotubes and nanofibers used or produced in U.S. facilities.**

Kelly Fraser^1,2^ ([yfd3@cdc.gov](mailto:yfd3@cdc.gov))*, Vamsi Kodali^1,2^ ([ywu0@cdc.gov](mailto:ywu0@cdc.gov))*, Naveena Yanamala^1,2^ ([yanamala.naveena@gmail.com](mailto:yanamala.naveena@gmail.com)), M. Eileen Birch^3^ ([mebirch1@gmail.co](mailto:mebirch1@gmail.co)m), Lorenzo Cena^4^ ([LCENA@wcupa.edu](mailto:LCENA@wcupa.edu)), Gary Casuccio^5^ ([gcasuccio@rjleegroup.com](mailto:gcasuccio@rjleegroup.com)), Kristin Bunker^5^ ([kbunker@rjleegroup.com](mailto:kbunker@rjleegroup.com)), Traci L. Lersch^5^ ([tlersch@rjleegroup.com](mailto:tlersch@rjleegroup.com)), Douglas E. Evans^3^ ([dje3@cdc.gov](mailto:dje3@cdc.gov)), Aleksandr Stefaniak^6^ ([boq9@cdc.gov](mailto:boq9@cdc.gov)), Mary Ann Hammer^1^ ([mcy2@cdc.gov](mailto:mcy2@cdc.gov)), Michael L. Kashon^1^ ([mqk1@cdc.gov](mailto:mqk1@cdc.gov)), Theresa Boots^1^ ([oph6@cdc.gov](mailto:oph6@cdc.gov)), Tracy Eye^1^ ([tmh7@cdc.gov](mailto:tmh7@cdc.gov)), John Hubczak^1,2^ ([odd6@cdc.gov](mailto:odd6@cdc.gov)), Sherri A. Friend^1^ ([shf8@cdc.gov](mailto:shf8@cdc.gov)), Matthew Dahm^7^ ([iwa6@cdc.gov)†](mailto:iwa6@cdc.gov)†), Mary K. Schubauer-Berigan^7,8^ ([BeriganM@iarc.fr)](mailto:BeriganM@iarc.fr)†) , Katelyn Siegrist^1,8^ ([kate.siegrist1@gmail.com](mailto:kate.siegrist1@gmail.com)), David Lowry^1^ ([dhl5@cdc.gov](mailto:dhl5@cdc.gov)), Alison K. Bauer^9^ (alison.bauer@cuanschutz.edu), Linda M. Sargent^1^ ([lindamsargent@outlook.com](mailto:lindamsargent@outlook.com)), Aaron Erdely^1,2^ ([efi4@cdc.gov](mailto:efi4@cdc.gov))

^1^Health Effect Laboratory Division, National Institute for Occupational Safety and Health, Morgantown, WV; ^2^West Virginia University, Morgantown, WV; ^3^Health Effects Laboratory Division, National Institute for Occupational Safety and Health, Cincinnati, OH; ^4^West Chester University, West Chester, PA; ^5^RJ Lee Group, Monroeville, PA; ^6^Repiratory Health Division, National Institute for Occupational Safety and Health, Morgantown, WV; ^7^Division of Field Studies Evaluation, National Institute for Occupational Safety and Health, Cincinnati, OH; ^8^International Agency for Research on Cancer, Lyon, France; ^9^University of Colorado Anschutz Medical Campus, Department of Environmental and Occupational Health, Aurora, CO

*These authors contributed equally to this work

**Corresponding author:** Aaron Erdely, PhD

NIOSH/HELD/PPRB

1095 Willowdale Rd, MS-2015

Morgantown, WV 26505-2888

Tel: 304-285-5903

Fax: 304-285-5708

e-mail: [efi4@cdc.gov](mailto:efi4@cdc.gov)


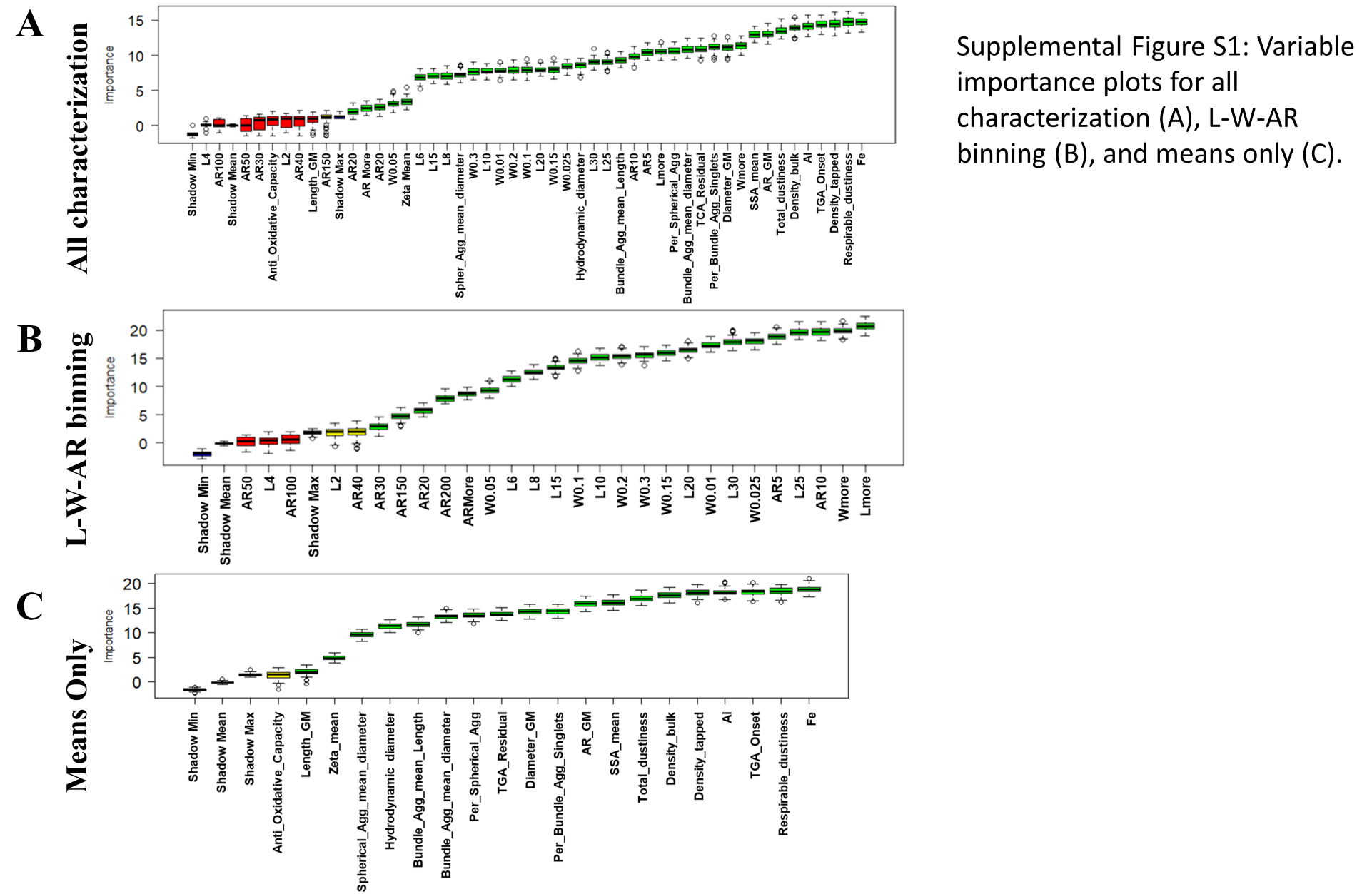


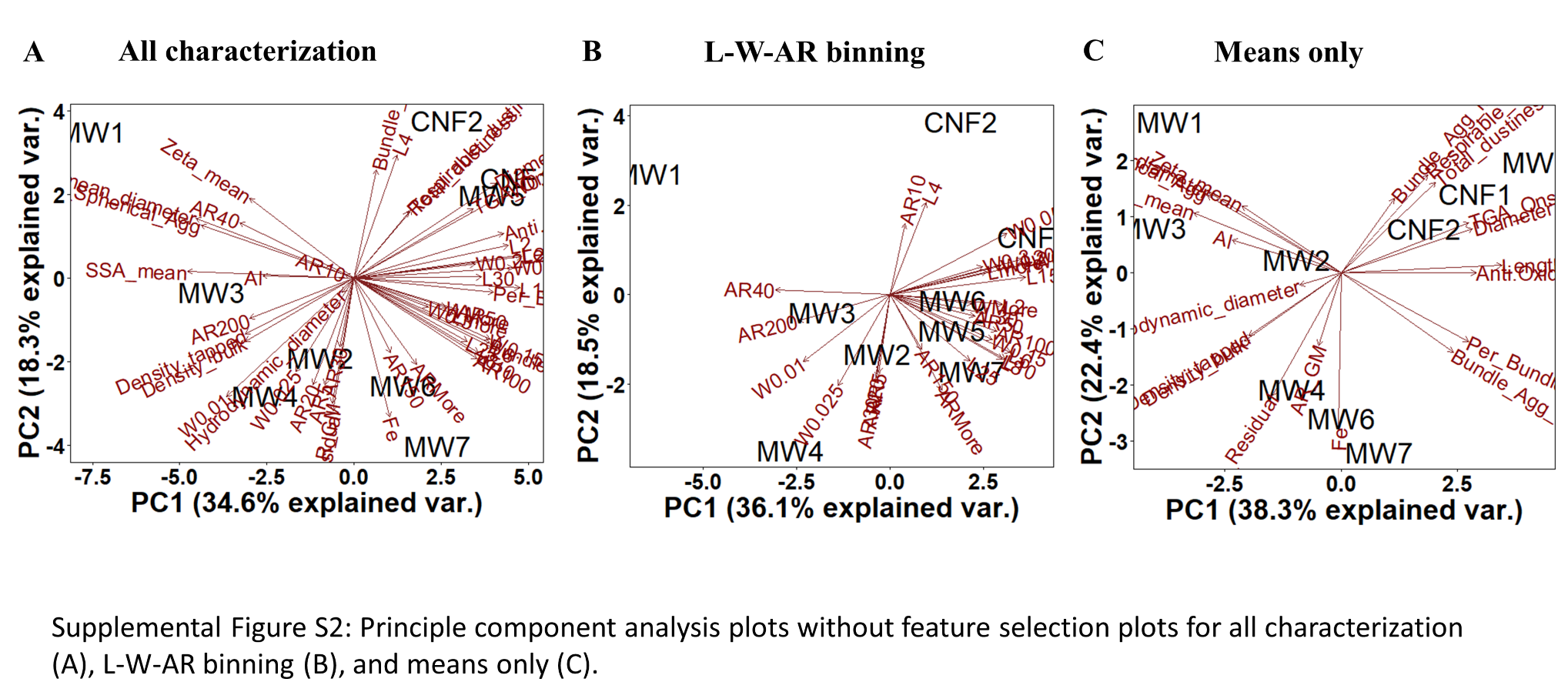


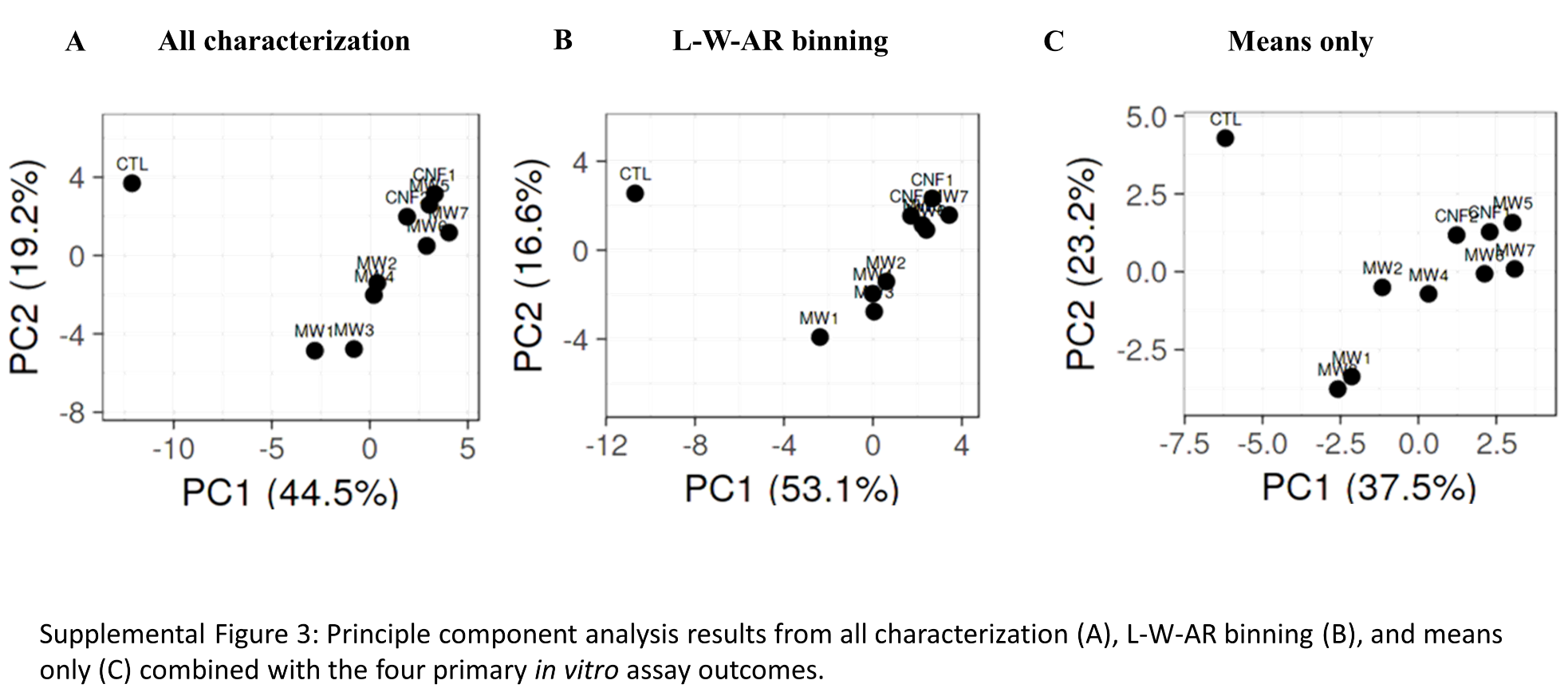


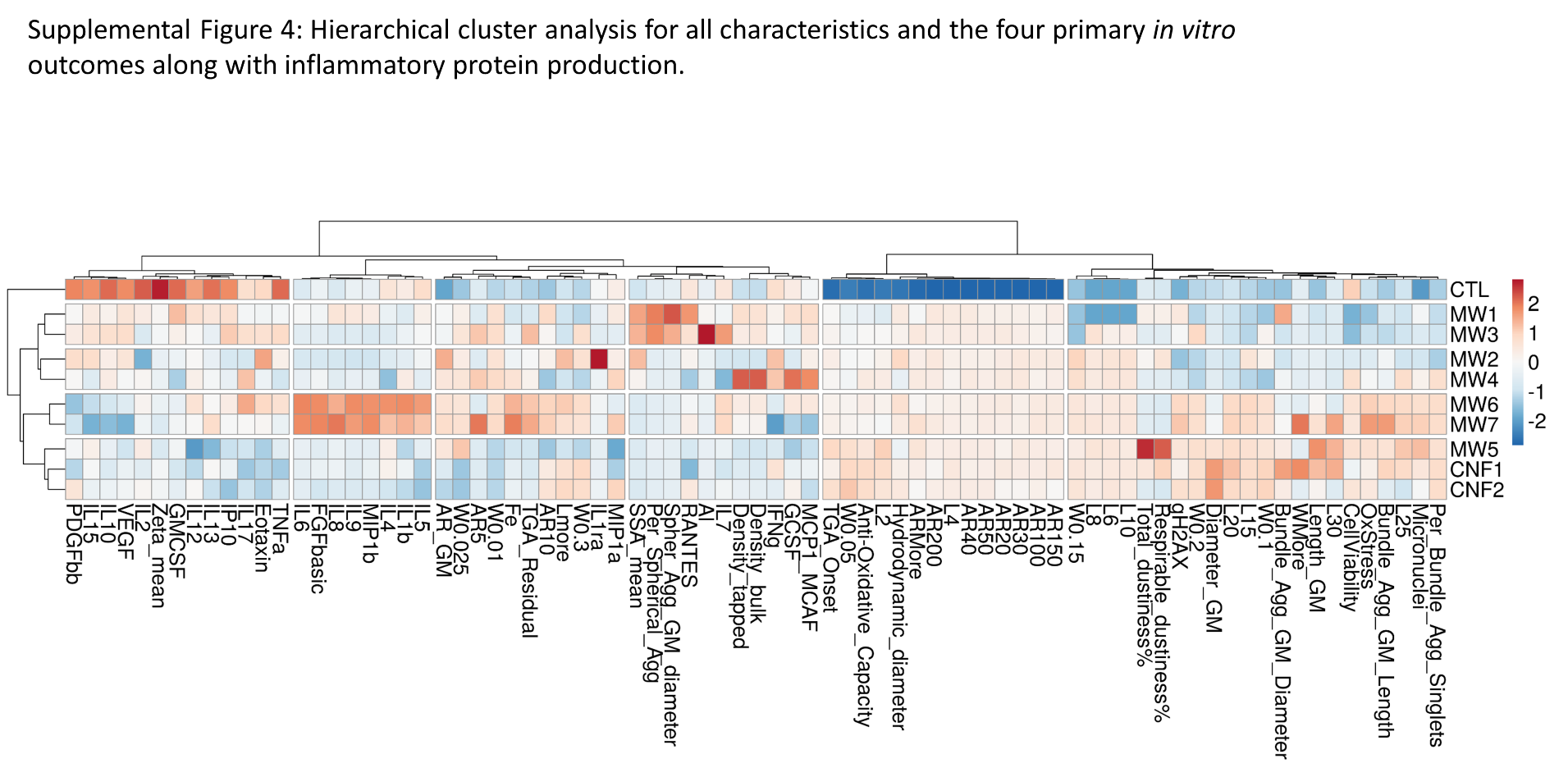


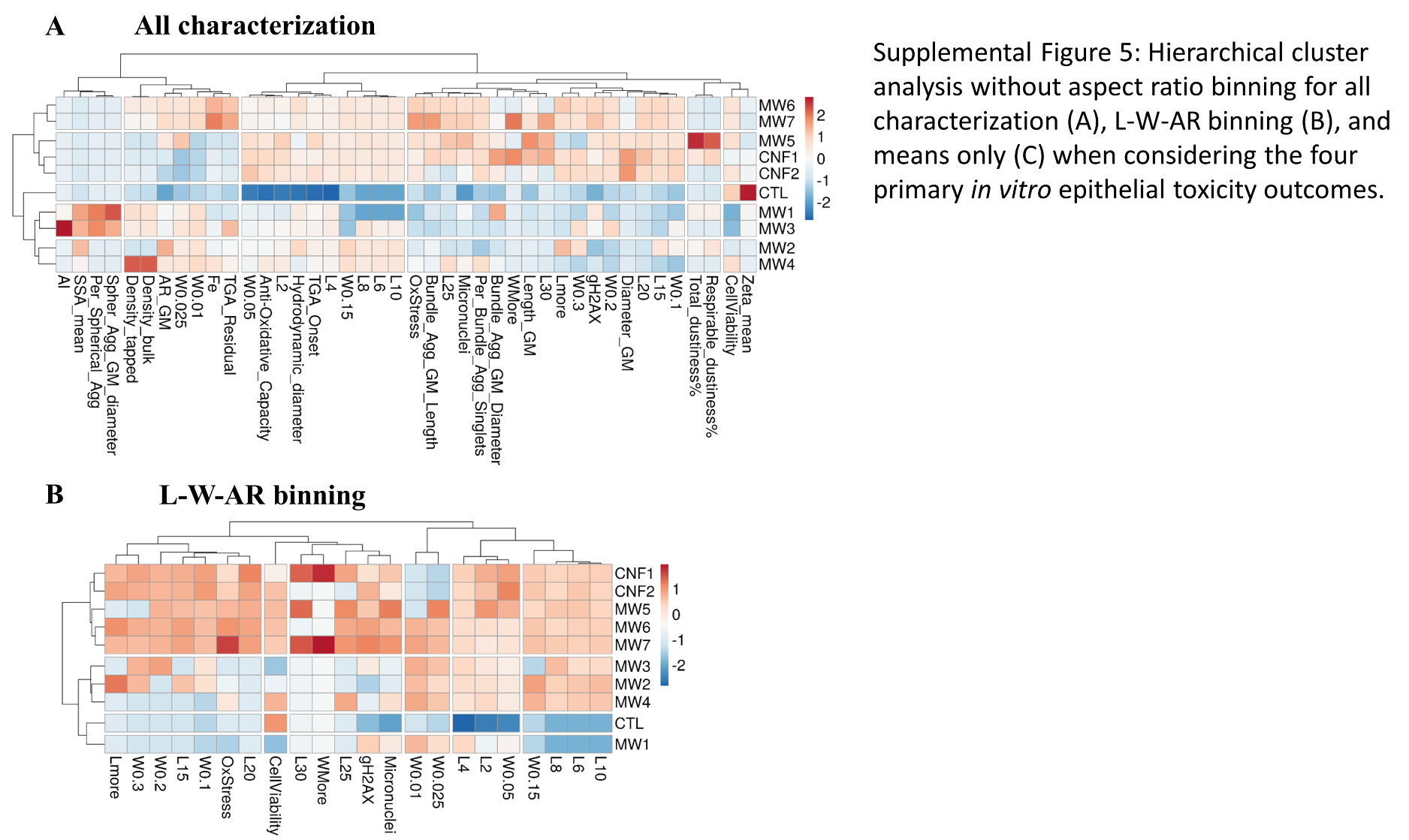


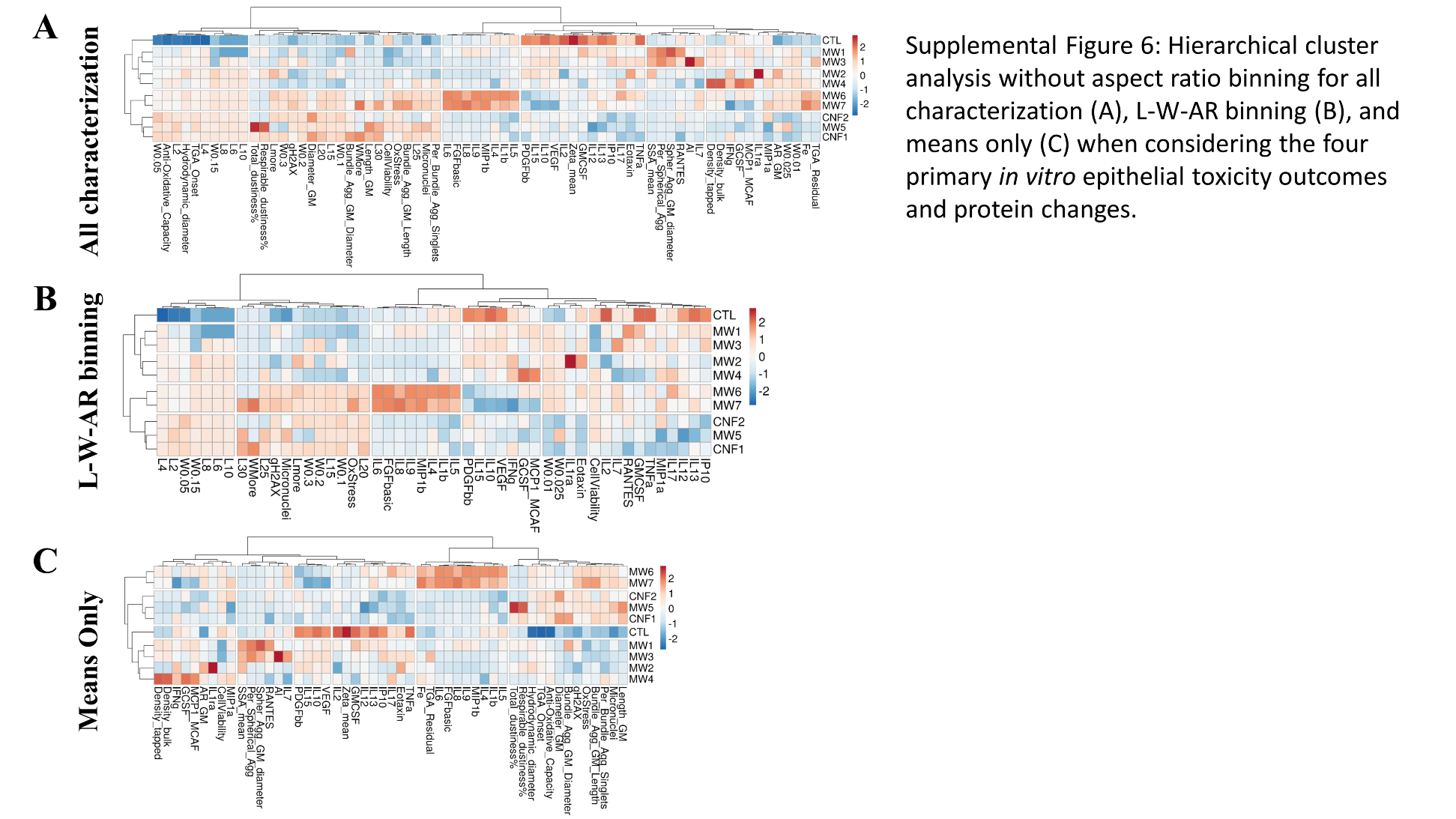


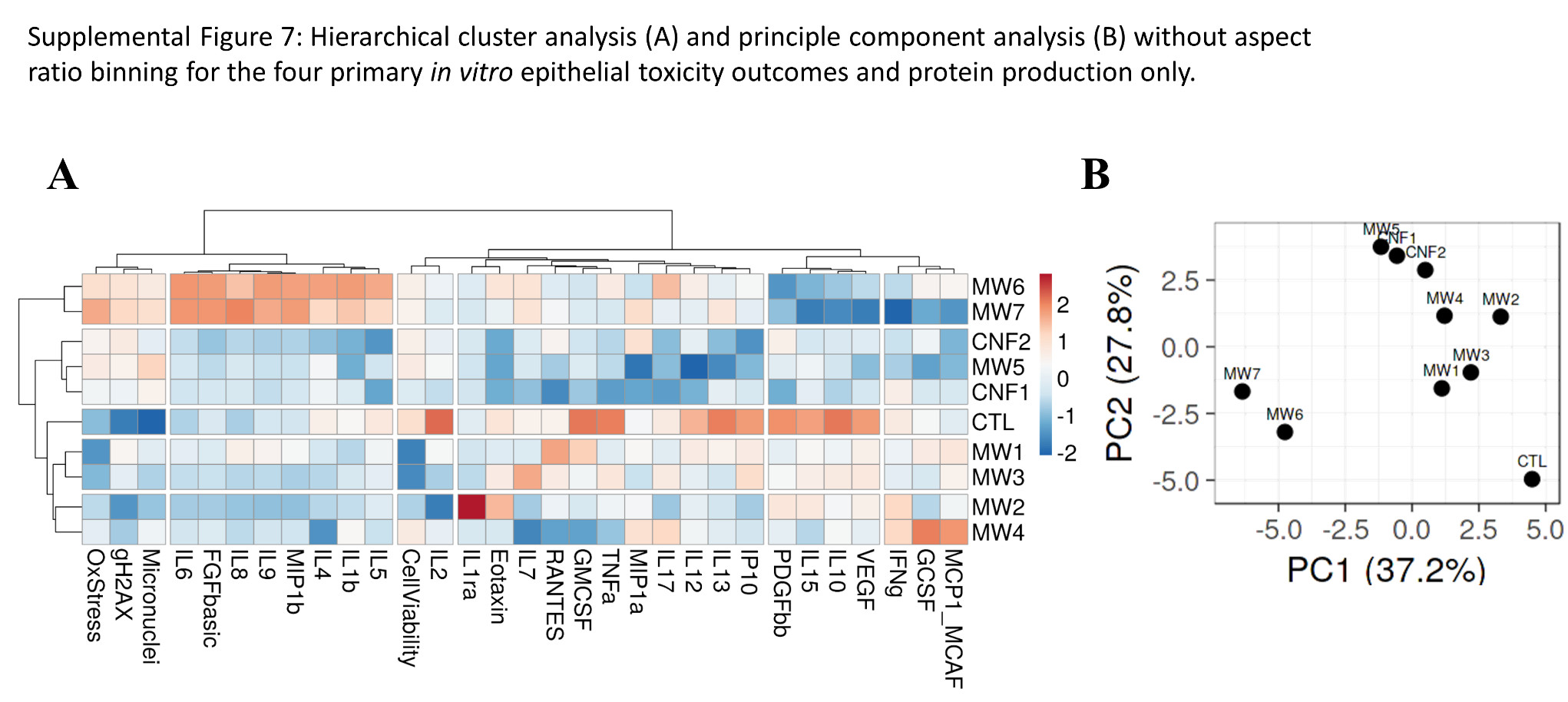


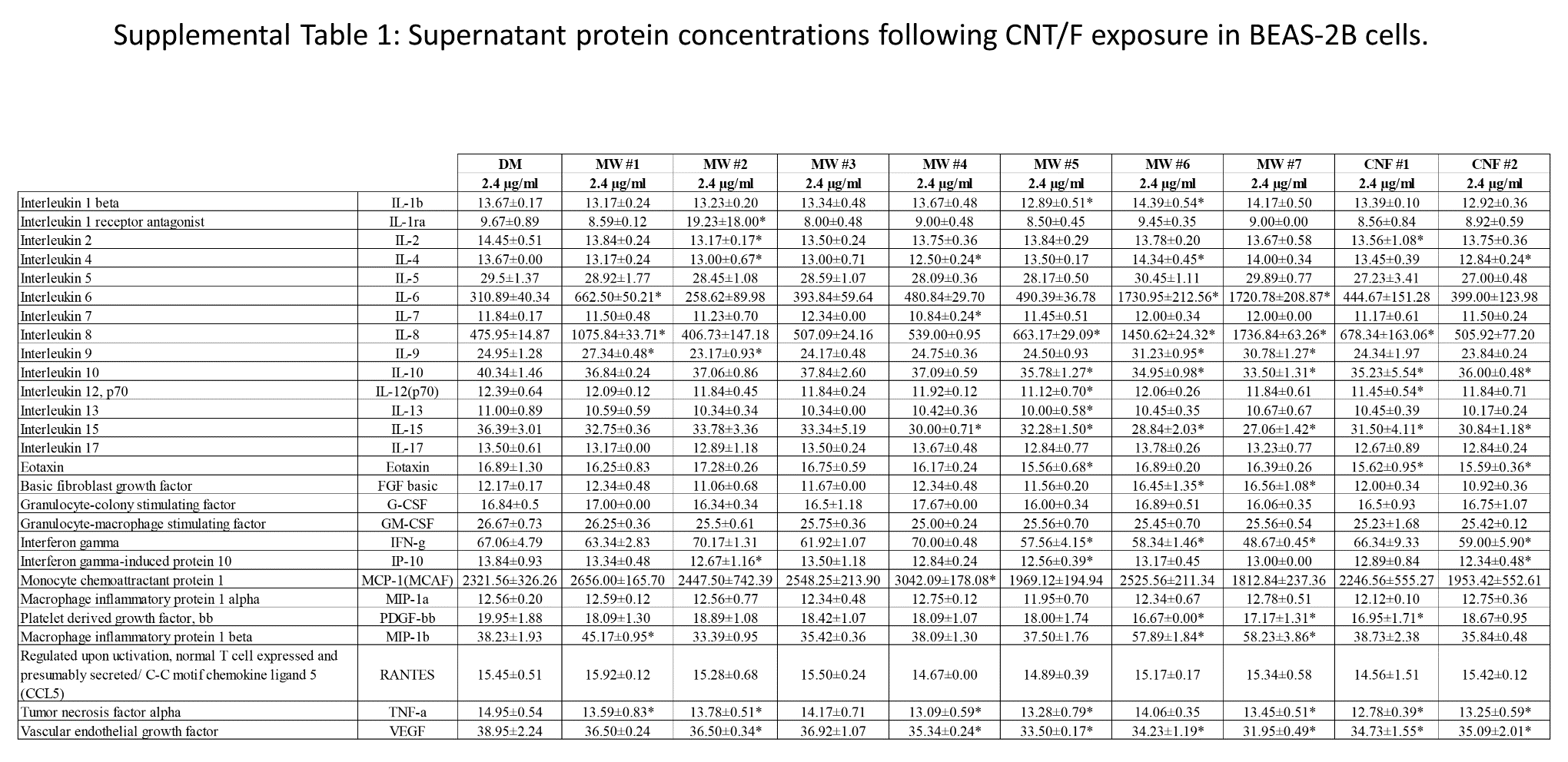


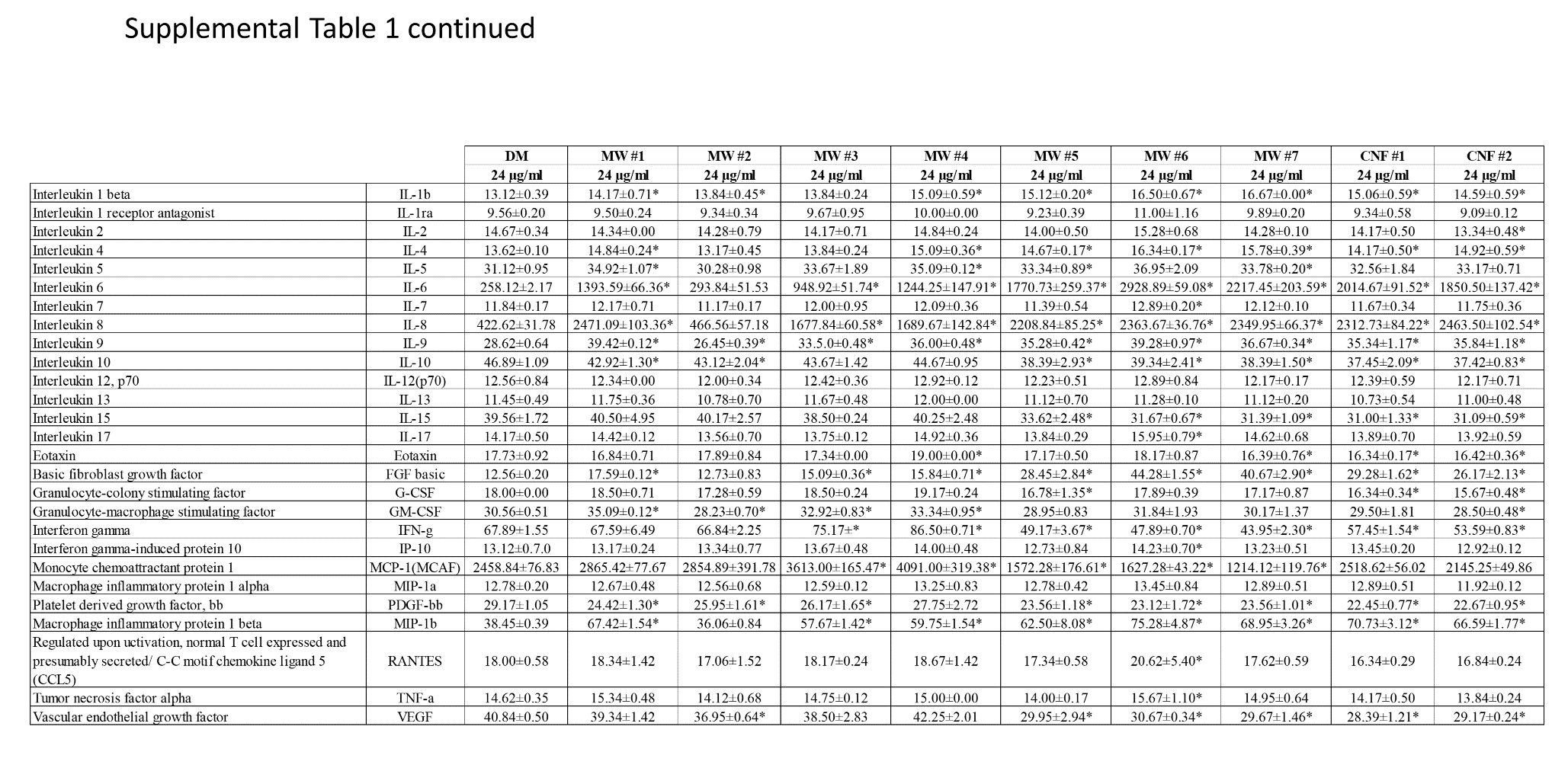

Supplement: Supplementary file 1 — Additional file 1. [file 12989_2020_392_MOESM1_ESM.docx]
